# Beyond the Beauty: Meristic and Genomic Signatures of Bangka’s Endemic Betta Fishes

**DOI:** 10.12688/f1000research.174779.2

**Published:** 2026-06-01

**Authors:** Helmizuryani Helmizuryani, Saleh Hidayat, Muhammad Nizar, Andhika Prima Prasetyo, Boby Muslimin, Mochammad Zamroni, Dessy Nurul Astuti, Swarlanda Swarlanda, Destra Ramadhanu, Luthfi Nurhidayat

**Affiliations:** 1 Study Program of Aquaculture, Faculty of Agriculture, Universitas Muhammadiyah Palembang, Palembang, South Sumatra, 30263, Indonesia; 2 Study Program of Biology Education, Faculty of Teacher Training and Education, Universitas Muhammadiyah Palembang, Palembang, South Sumatra, 30263, Indonesia; 3 Research Center for Applied Zoology, National Research and Innovation Agency, Cibinong, West Java, 16911, Indonesia; 4 Research Center for Biota System, National Research and Innovation Agency, Cibinong, West Java, 16911, Indonesia; 5 Yayasan Ikan Endemik Bangka Belitung, Pangkal Pinang, Bangka Belitung Island, 33123, Indonesia; 6 Faculty of Biology, Universitas Gadjah Mada, Sleman, Special Region of Yogyakarta, 55281, Indonesia

**Keywords:** Betta burdigala, Betta chloropharynx, Betta schalleri, meristic analysis, genome assembly, synteny, conservation genomics, Bangka Island

## Abstract

**Background:**

The genus *Betta* (family Osphronemidae) comprises over 70 species, many of which are endemic to Southeast Asia and highly vulnerable to habitat loss. While *Betta splendens* is well studied due to its importance in the ornamental fish trade, most wild *Betta* species remain poorly characterized, particularly at the genomic level. The Bangka Islands of Indonesia harbor several endemic *Betta* species threatened by peatland degradation.

**Methods:**

We conducted an integrated meristic and genomic comparison of three endemic Bangka Island *Betta* species— *Betta burdigala*, *B. chloropharynx*, and *B. schalleri.* Specimens were collected from peatland waters in Bangka, and meristic traits were examined to confirm diagnostic characteristics. High-molecular-weight DNA was extracted and sequenced using Oxford Nanopore PromethION technology, followed by *de novo* assembly and reference-guided scaffolding using the *Betta splendens* genome.

**Results:**

The meristic analysis confirmed features consistent with their taxonomic placement within the *coccina*, *waseri*, and *pugnax* groups. Genome assemblies were highly contiguous and complete (BUSCO >97%), with *B. chloropharynx* showing the largest genome size, highest scaffold N50, and elevated retrotransposon content. Gene duplication analysis revealed dispersed duplications as the dominant category across all genomes, with variation in tandem and proximal duplicates. Comparative genomic analysis demonstrated conserved collinearity, with *B. chloropharynx* and *B. schalleri* showing the closest relationship, while *B. burdigala* diverged earlier. The Colony Stimulating Factor 1 Receptor A (CSF1RA) protein phylogenetic tree closely resembles the phylogenetic tree of nine fish species based on NCBI taxonomic data. We also identified two massive protein insertions in the CSF1RA of *B. burdigala.*

**Conclusions:**

This study provides morphological and genomic evidence supporting the distinctiveness of Bangka’s endemic *Betta* species and delivers essential genomic resources for evolutionary research and conservation of these endangered freshwater fishes.

## Introduction

The genus *Betta* (family Osphronemidae) includes over 70 recognized species, many of which are endemic to Southeast Asia. Despite the global popularity of the domesticated Betta splendens in the ornamental fish trade, the majority of wild Betta species remain under-researched. Indonesia harbors nine wild *Betta* species classified as Critically Endangered (CN) and seven species categorized as Endangered (EN). ^1– 3^ In comparison, Thailand contains two Critically Endangered (CE) wild *Betta* species and one Endangered (EN) species. ^4, 5^ The high levels of endemism and restricted ranges of many Betta species make them particularly vulnerable to habitat loss and environmental changes. ^2, 5^

The Bangka Islands, located off the east coast of Sumatra, are home to several unique Betta species adapted to specialized freshwater habitats such as peat swamps and slow-moving forest streams. Three *Betta* species— *B. burdigala*, *B. chloropharynx*, and *B. schalleri*—are endemic to the Bangka Islands. ^1^ According to IUCN Red List, the conservation status for them were Critically Endangered (CR) and Endangered (EN). The lack of molecular resources poses a barrier to implementing effective conservation strategies for these fishes. Previous studies on the endemic *Betta* species from Bangka Island have included genetic analyses based on mitochondrial DNA and eDNA metabarcoding, as well as comprehensive morphological examinations, all of which have contributed to clarifying the genetic relationships and taxonomic status of these species. However, the whole-genome evolutionary landscape of the endemic *Betta* species from Bangka Island has not yet been explored. ^6^

Genome assemblies are indeed foundational resources for advancing biological research and conservation management. They provide critical insights into genetic diversity, evolutionary biology, and species conservation strategies. High-quality genomic data could be used for Long-read sequencing and high-throughput chromosome (Hi-C) technology. Long-read sequencing technologies are essential for producing high-quality genome assemblies. High-quality genomic data can be generated using long-read sequencing technologies such as Oxford Nanopore sequencing, which enables the production of highly contiguous genome assemblies. These technologies help resolve complex repeats and haplotype heterozygosity, which are sources of assembly errors. ^7^ Genome assemblies could be informative evidence for scientific management decisions and tools for understanding the basis of genetic adaptation in various species. ^8, 9^

Bangka Island presents a unique opportunity to investigate the genetic foundations of adaptation and diversity in insular freshwater fish species. The endemic Betta species of the island have likely undergone distinct evolutionary trajectories due to the island’s unique environmental conditions and geographic isolation. Producing genome assemblies for these taxa will address a significant knowledge gap and serve as a foundational step for future conservation genomics efforts in the region. This study aims to generate preliminary genome assemblies for three endemic *Betta* species originating from Bangka, Indonesia, utilizing whole-genome sequencing methodologies.

## Materials and methods

### Ethics statement

This study received ethical approval from the Ethics Commission for Animal Care and Use, National Research and Innovation Agency Republic of Indonesia (Approval No. 220/KE.02/SK/09/2024). All animal procedures followed institutional regulations and adhered to the ARRIVE 2.0 reporting guidelines, with the corresponding checklists available at https://doi.org/10.6084/m9.figshare.31136455. ^10^

### Sample collection

**Figure 1.**
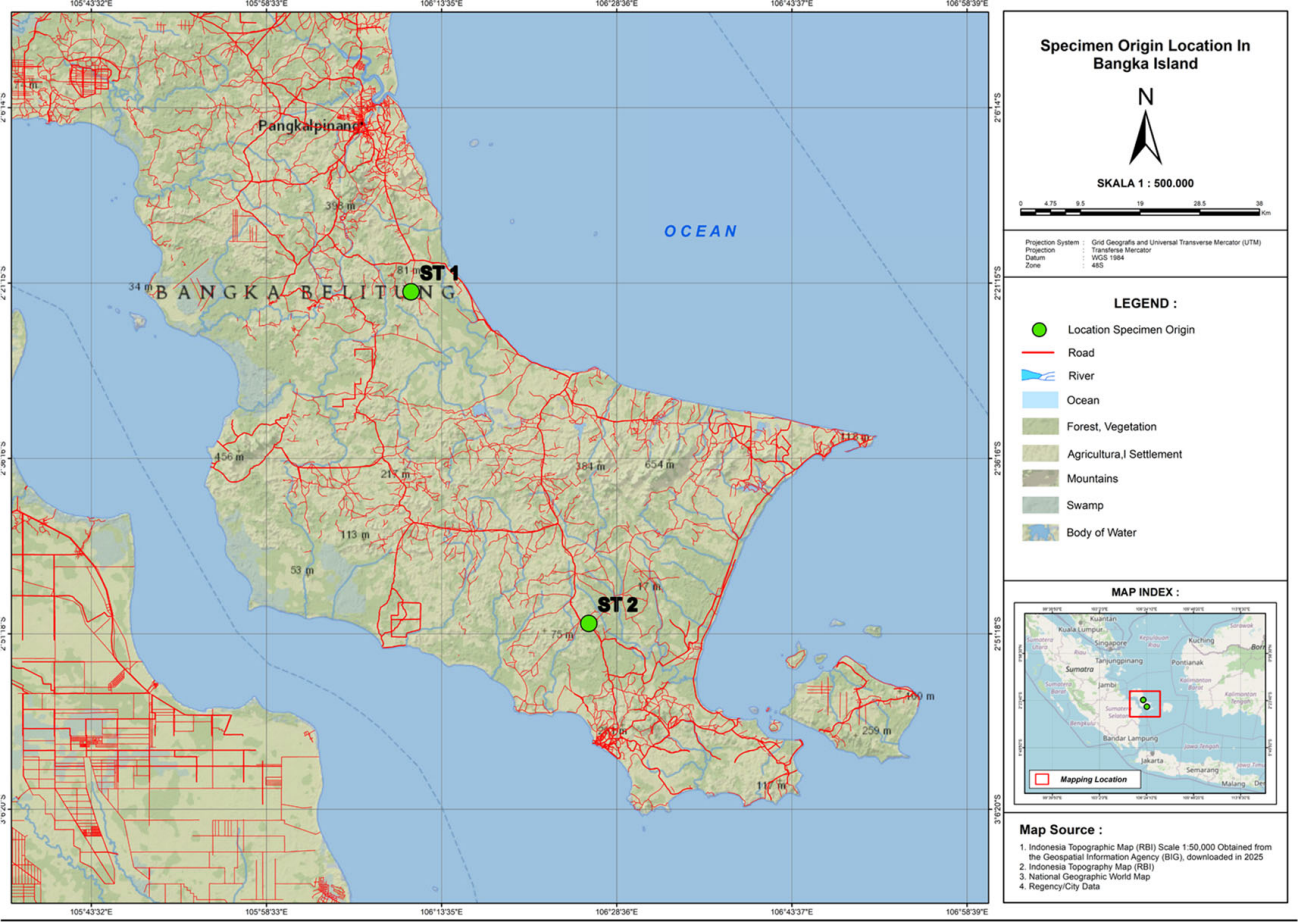
The specimen origin locations of *Betta schalleri* and *Betta chloropharynx* are found in ST.1 (Central Bangka), while *Betta burdigala* is found in ST.2 (South Bangka).

This study examines three endemic species of Betta: *Betta burdigala*, *Betta chloropharynx*, and *Betta schalleri.* The fish samples were collected from peatland water in South Bangka, Bangka Island, Indonesia. The map showing the origin locations of specimens on Bangka Island is presented in Figure 1. The males of *B. schalleri* and *B. chloropharynx* have been found in Central Bangka (2°22'00.7"S 106°10'57.9"E) and *B. burdigala* has been found in South Bangka (2°50'24.8"S 106°26'12.7"E). The specimens were collected using a scope net with a mesh size of 0.5 mm as active gear. In-situ sampling data for morphometric and meristic analysis were obtained from three individuals each of *B. schalerii*, *B. chloropharynx*, and *B. burdigala.* The ten morphometric and seven meristic characters (Suppl Fig 1) based on Nur et al. (2022) ^2^ were measured using a digital caliper with an accuracy 0.1 mm. The sampling design in this study used purposive sampling based on mature females or males with different shapes and colors. Males have longer ventral fins than females. ^11^ The males also have more vibrant and diverse body colors than females. ^12^ The morphometric and meristic data were analyzed descriptively.

One male specimen from each Betta species was euthanized using the rapid chilling method, which involved transferring the fish to water maintained at approximately 2°C with crushed ice. ^13^ The fish was left in this condition for 20 minutes until opercular movement ceased. Subsequently, tissue samples were collected, fixed with absolute ethanol, and stored in cryotubes for whole genome sequencing at the Central Sequencing Laboratory, BRIN, Bogor, Indonesia.

### Genomic DNA extraction and sequencing

The extraction of genomic DNA was performed using the Applied Biosystem MagMax ^TM^ DNA Multi-Sample 2.0 kit (Thermo Fisher Scientific; CAT. A36570) following the manufacturer’s instructions. Twenty mg of fish tissue is used for genome DNA extraction material for all Betta specimens. The genome concentration from the extraction was checked using Nanodrop and Qubit. Genome concentration by Nanodrop value between 227.45-289 ng/μl and Qubit value between 89.4-91.6 (ng/μl). The genome quality was checked using gel agarose with TBE agarose concentration 1%. The genomic DNA was subsequently prepared for library construction using the Ligation Sequencing DNA V14 kit (Oxford Nanopore Technology; SQK-LSK114) in accordance with the manufacturer’s instructions. Following this, the library was sequenced on a PromethION device utilizing PromethION Flow Cells Packs (Oxford Nanopore Technology; FLO-PRO114M). The sequencing parameters we employed included a run duration of 96 hours, a pre-scan interval of 1.5 hours, basecalling using the High-accuracy model at 400 bps, and a minimum Q score of 9. The sequencing software utilizes MinKNOW (25.03.7), Bream (8.4.4), configuration (6.4.10), basecalling Dorado (dna_r10.4.1_e8.2_ 400bps_hac@4.3.0), and MinKNOW Core (6.4.8). The genome sequence data for *B. burdigala* comprised 16.38 million reads totaling 37.39 Gb, with a read N50 of 5.23 kb, corresponding to an estimated genome coverage of approximately 74.8–83.1× assuming a genome size of 450–500 Mb. For *Betta chloropharynx*, sequencing generated 14.97 million reads totaling 57.97 Gb, with a read N50 of 7.75 kb, representing an estimated coverage of approximately 115.9–128.8×. Meanwhile, *Betta schalleri* produced 11.83 million reads totaling 23.75 Gb, with a read N50 of 5.5 kb, corresponding to an estimated genome coverage of approximately 47.5–52.8×.

### Genome assembly and annotation

The genome assembly and annotation described in this paper were conducted in accordance with the methodology performed by Imron et al. ^14^ Unless otherwise stated, all software tools were run using their default parameters. Adapter contamination screening was performed using Porechop_ABI prior to assembly. ^15^ Genome assembly was done using Flye 2.9.5, ^16^ while genome scaffolding was conducted using RagTag 2.1.0 ^17^ guided by genome reference of *Betta splendens* (GCF_900634795.4). Foreign contamination was screened and removed using NCBI FCS-GX prior to scaffolding. ^18^ Genome size estimation was conducted using Jellyfish software version 2.3.1 ^19^ and was further processed using GenomeScope 2.0 v2.0.1. The assembly statistics were calculated using assembly stat version 1.0.1. The completeness of the assembly was estimated using Benchmarking Universal Single-Copy Orthologous (BUSCO) version 5.8.2, utilizing miniprot ^20– 22^ with the Actinopterygii lineage dataset.

Repetitive elements within the genome assembly were identified using RepeatModeler v2.0.6 in conjunction with RepeatMasker v4.1.7. ( http://www.repeatmasker.org). Prior to annotation, these repetitive regions were soft masked to minimize interference. Structural genome annotation encompassing gene prediction was conducted using the GALBA pipeline, ^23^ which employs miniprot ^22^ and AUGUSTUS, ^24^ integrating protein data from closely related species as extrinsic evidence. Specifically, protein data from *Betta splendens* (GCF_900634795.4), *Anabas testudineus* (GCF_900324465.2), and *Channa argus* (GCF_033026475.1) were utilized. Functional annotation of the resulting gene predictions was then performed using the ‘funannotate annotate’ command from the Funannotate pipeline ( https://funannotate.readthedocs.io/en/latest/install.html), incorporating tools such as InterProScan5, ^25^ Eggnog-Mapper, ^26^ and SignalP 5.0 ^27^ to assign gene names and predict protein functions. Finally, the completeness of the genome annotation was evaluated using BUSCO v5.8.2. ^21^ with the Actinopterygii lineage dataset. For annotation-level assessment, BUSCO analysis was performed on the complete predicted protein dataset generated from the annotation pipeline, including all annotated transcript isoforms rather than restricting the analysis to only the longest isoform per gene.

### Duplicate genes classification and comparative genomics

The protein sequences in each genomes were aligned to eachother using BLAST+ v. 2.14.1 ( https://blast.ncbi.nlm.nih.gov/doc/blast-help/downloadblastdata.html). The alignment results and the gff (general feature format) file containing gene position in the genome were then further analyzed using the ‘Duplicate_gene_classifier’ function of MCScanX ^28^ to identify gene duplications.

We then performed all-against-all alignment of protein sequences of three Betta genomes: using BLAST+ v. 2.14.1 ( https://blast.ncbi.nlm.nih.gov/doc/blast-help/downloadblastdata.html). The analyses were performed using an E-value threshold of 1e−3, with results limited to a maximum of five target hits per query sequence and output generated in tabular format (outfmt 6). Computations were executed using 12 parallel threads, while all other parameters were retained at their default settings. The alignment results were then concatenated into one file. The gff files were also concatenated. These files were then further analyzed using MCScanX ^28^ to detect gene synteny and collinearity. The results were then explored and visualize using SynVisio. ^29^

We also performed comparative analysis of Colony Stimulating Factor 1 Receptor A (CSF1RA), Melanocortin 1 Receptor (MC1R) and Paired Box 7 (PAX7) protein sequence among *B. burdigala*, *B. chloropharynx*, and *B. schalleri*, alongside *B. splendens, Anabas testudineus, Channa argus, Oreochromis niloticus, Danio rerio, and Carassius auratus.* The protein accession of CSF1RA that we used are: XP026135817.1, XP026135818.1, NP571747.1, XP021336731.1, XP003455234.1, XP013133007.1, XP067373776.1, XP067373775.1, XP026213579.1, XP055368253.1, XP055368253.1, and XP029020690.1. The protein accession of MC1R that we used are: NP_851301.1, XP_005159236.1, XP_026112973.1, XP_026112974.1, XP_005467175.1, XP_055363428.1, XP_067343026.1, XP_067343026.1, XP_026234205.1. The protein accession of PAX7 that we used are: XP_025763203.1, XP_005459058.1, XP_067361857.1, XP_029005486.1, XP_029005485.1, XP_026204402.1, XP_026204405.1, XP_025763202.1, XP_067361858.1, XP_026204404.1, XP_029005487.1, XP_026204403.1, NP_571407.2, NP_571400.1, XP_009304561.1, XP_026130952.1, XP_026130953.1. The protein sequence alignments were performed using Clustal Omega ^30^ via the European Bioinformatics Institute website ( https://www.ebi.ac.uk/jdispatcher/msa/clustalo). Subsequently, these alignments were used for phylogenetic reconstruction with IQ-TREE, ^31^ and the results were visualized using TreeViewer. ^32^

## Results

### Fish morphology

**Figure 2.**
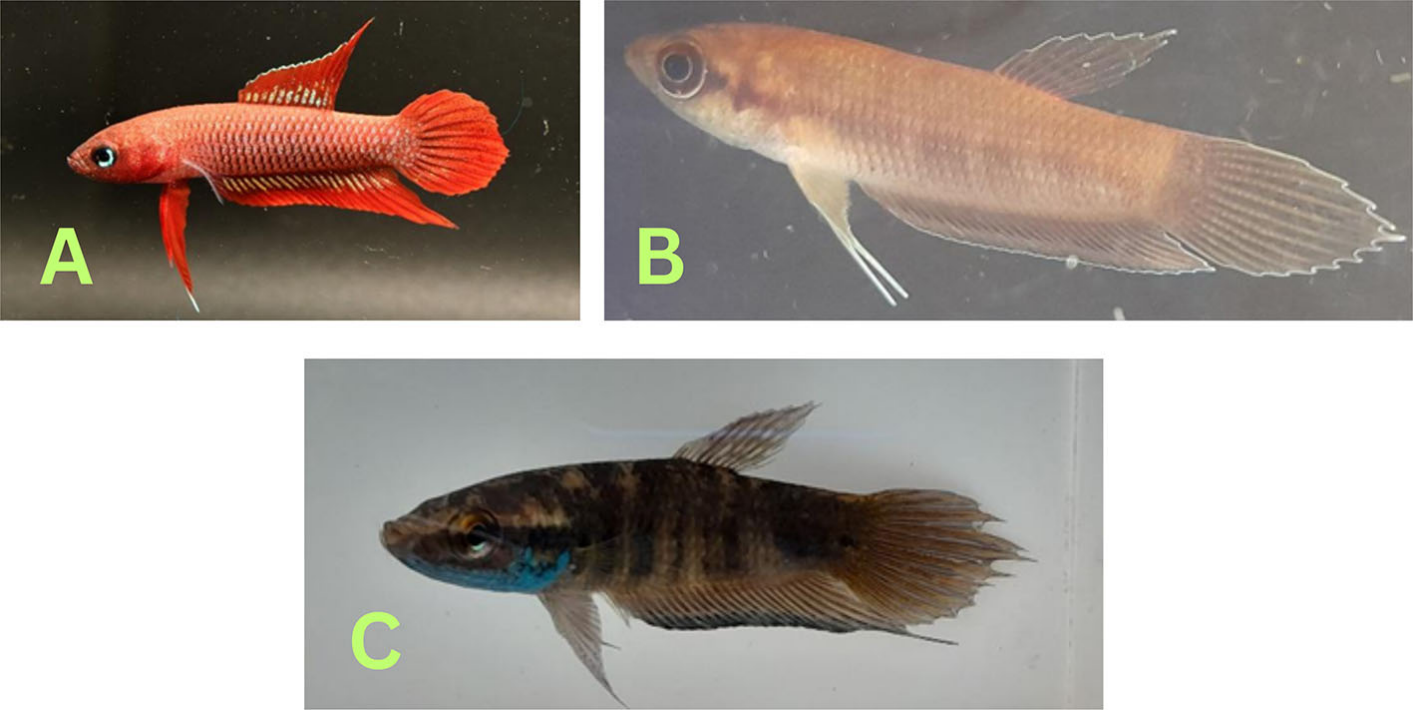
The morphology of *B. burdigala* (A), *B. chloropharynx* (B) and *B. schalleri* (C).

The morphology photo of Betta spp. is presented in this research as shown in Figure 2 for *B. burdigala* (A), *B. chloropharynx* (B), and *B. schalleri* (C). Table 1 outlines the meristic characteristics that distinguish them. The meristic and morphometric characters are presented in Suppl Fig 1.

**Table 1.** Meristic and morphometric character of *B. burdigala, B. chloropharynx*and and *B. schalleri.* The average of morphometric characters as a percentage to standard length with standard deviation.

	*B. burdigala*	*B. chloropharynx*	*B. schalleri*
**Meristic character**			
Dorsal fin radii	D.I. 13–14	D.I.8-9	D.I.8-9
Anal fin radii	A.I–II. 23–24	A.I-II. 27-28	A.I-II.22-23
Caudal radii	C.XV	C.XV	-
Standard length (cm)	2.46 ± 0.13	9.95 ± 0.91	5.21 ± 1.23
**Morphometric character**			
Post-orbital length (%)	34.71 ± 8.17	47.60 ± 1.06	45.52 ± 0.74
Dorsal fin length (%)	30.43 ± 1.68	14.00 ± 0.29	15.01 ± 0.95
Predorsal length (%)	51.96 ± 2.20	65.17 ± 0.81	63.01 ± 1.26
Postdorsal length (%)	17.33 ± 3.72	22.45 ± 1.69	21.16 ± 1.28
Orbital diameter (%)	6.52 ± 1.81	9.63 ± 0.51	9.24 ± 0.75
Body depth (%)	20.48 ± 1.28	25.78 ± 1.68	27.59 ± 0.25
Head length (%)	29.04 ± 0.60	33.97 ± 1.40	34.90 ± 1.90
Anal fin length (%)	46.15 ± 5.16	49.30 ± 0.17	47.73 ± 1.91
Preanal length (%)	47.73 ± 3.99	47.45 ± 1.16	47.91 ± 0.53

### Betta burdigala

*Betta burdigala* is classified within the *Betta coccina* group. ^33^ This species is characterized by its red coloration and a size range of 2-3 cm, with distinct morphological features illustrated in Figure 2A that differentiate it from other species within the *Betta coccina* group. The meristic characteristics of *B. burdigala* are comprehensively presented in Table 1. In comparison to *B. uberis*, ^34^ *B. burdigala* exhibits fewer dorsal fin rays (14-15 vs. 14-17), subdorsal scales (11-11.5 vs. 12-13.5), predorsal scales (15-16 vs. 18-20), and postdorsal scales (8 vs. 9-11). Regarding morphometric characteristics, *B. burdigala* demonstrates a longer postdorsal length as a percentage of standard length (12.19-20.85% vs. 13.5-17.8%), a shorter dorsal fin base (28.12-32.03% vs. 30.0-37.2%), and a greater postorbital length (23.20-41.36% vs. 13.4-16.8%).

### Betta chloropharynx

*Betta chloropharynx*, depicted in Figure 2B, is a member of the *Betta waseri* group. ^35^ This group is distinguished by a unique pattern on the ventral side of the head. *Betta chloropharynx* can be differentiated from *B. hipposideros* by several characteristics: it possesses ω-shaped black throat markings as opposed to horseshoe-shaped ones, lacks transverse lines on the caudal fin, and has fewer subdorsal scales, typically 5-6 compared to 6.5 ( Table 1). In comparison to *B. renata*, *B. chloropharynx* exhibits ω-shaped black throat marks instead of kidney-shaped ones, lacks transverse lines on the dorsal and caudal regions, has an unspotted operculum rather than a speckled one, and features a yellow underside on the operculum instead of a black edge, with fewer subdorsal scales, mode 5-6 versus 6.5. The distinctions between *B. chloropharynx* and *B. spilotogena* include ω-shaped black throat marks versus a central black spot, an unspotted operculum versus a spotted one, and an operculum with a yellow underside versus a posterior edge. ^36^

### Betta schalleri

In Figure 2C, *Betta schalleri*, when compared to other *B. pugnax*, ^36^ demonstrates distinct meristic characteristics. It possesses a greater number of anal fin rays (27 vs. 23-25), a higher count of dorsal fin rays than *B. cracens* and *B. fusca* (10-11 vs. 8-9), more subdorsal scales than *B. cracens* and *B. fusca* (6.5-7 vs. 5.5-6), a greater number of lateral scales than *B. fusca* (31 vs. 29), fewer lateral scales than *B. cracens* (31 vs. 32-33), and a reduced number of predorsal scales compared to *B. cracens, B. fusca,* and *B. raja* (17-19 vs. 20-24). In term of morphometric characteristics (expressed as a percentage of standard length; Table 1), *B. schalleri* exhibits a longer head length compared to *B. pugnax*, *B. cracens,* and *B. fusca* (35.5-36.5% vs. 27.5-35.2%), a shorter predorsal length compared to *B. fusca* (62.7-66.3% vs. 68.5-70.2%), a longer preanal length compared to *B. cracens* (47.16-48.30 % vs. 42.0-46.1%), a greater body depth compared to *B. cracens* (27.38-27.93 % vs. 21.2-24.2%), an extended dorsal fin base compared to *B. cracens* (14.11-16.32% vs. 10.5-11.6%), and a shorter anal fin base compared to *B. cracens* (45.49-50.16 % vs. 53.4-55.7%).

### Genome assembly

**Figure 3.**
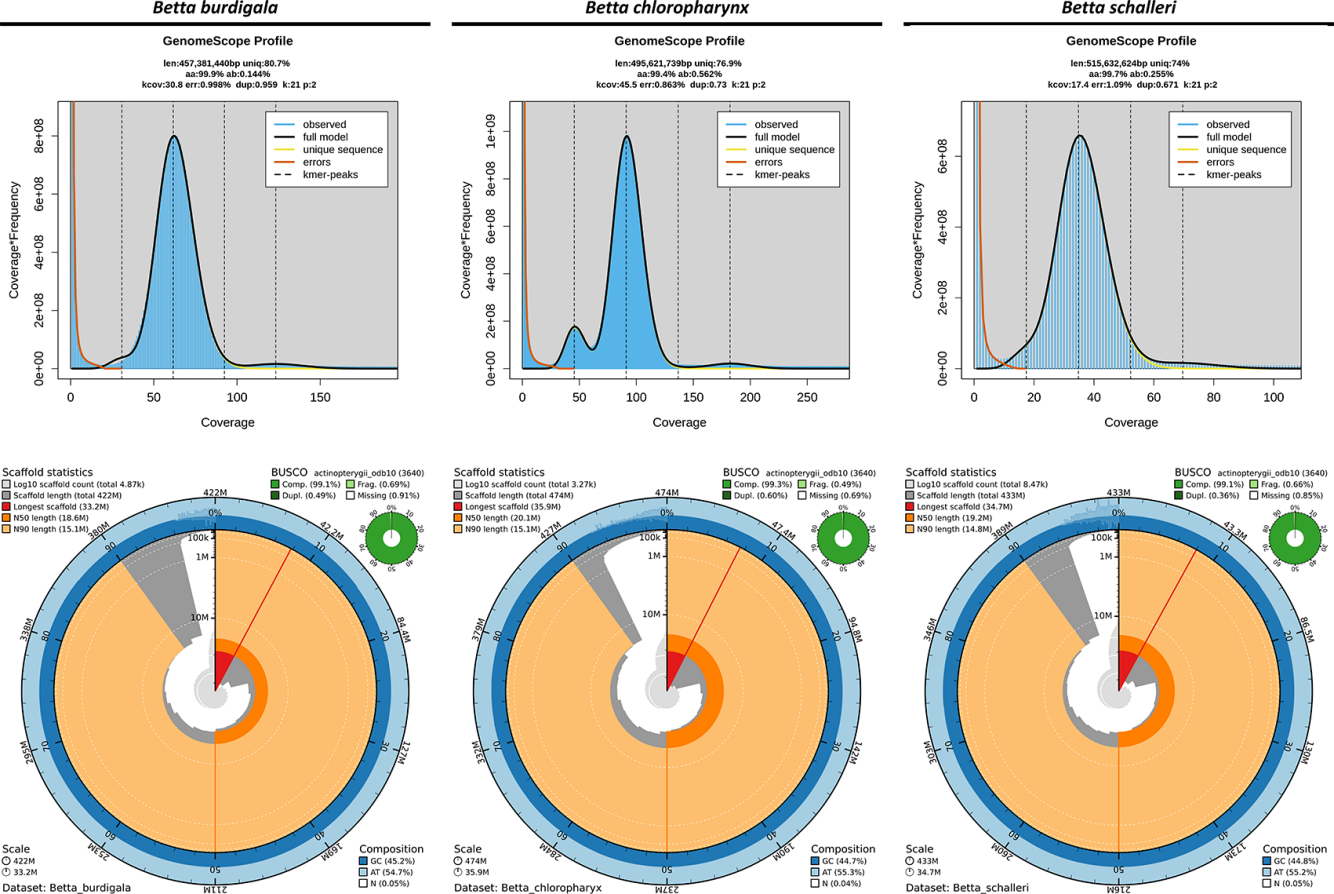
21-mer–based genome size estimation (upper panels) and summary of genome assembly characteristics (lower panels) for *B. burdigala*, *B. chloropharynx*, and *B. schalleri*.

The 21-mer profiling using GenomeScope indicated that the three endemic *Betta* species possess moderately compact genomes with relatively low heterozygosity levels, with *Betta schalleri* exhibiting the largest estimated genome size, followed by *Betta chloropharynx* and *Betta burdigala* ( Figure 3). The genome assemblies of *B. burdigala*, *B. schalleri*, and *B. chloropharynx* demonstrated high contiguity and completeness, with *B. chloropharynx* showing the strongest contiguity, while *B. burdigala* and *B. schalleri* also provide high-quality genomic resources suitable for comparative and evolutionary studies ( Figure 3). The genome assembly of *B. burdigala* totaled ~422 Mbp across 4,868 scaffolds and 6,904 contigs, with a scaffold N50 of 18 Mb and the longest scaffold reaching 33.2 Mb. The assembly was highly complete, with a BUSCO score of 99.1% (98.6% single-copy, 0.5% duplicated), while the GC content was 45.2% and gaps accounted for only 0.048%. The *B. chloropharynx* genome was slightly larger at ~474 Mbp, assembled into 3,273 scaffolds and 4,918 contigs. It showed the greatest contiguity, with a scaffold N50 of 20 Mbp, a maximum scaffold length of 35.9 Mbp, and the highest mean scaffold length (~145 kb). BUSCO completeness reached 99.3% (98.7% single-copy, 0.6% duplicated), the highest among the three species, with minimal fragmentation (0.5%) and missing genes (0.2%). The *Betta schalleri* assembly spanned ~433 Mbp with 8,474 scaffolds and 10,762 contigs, reflecting greater fragmentation. The scaffold N50 was 19 Mb, with the longest scaffold at 34.7 Mbp and a mean length of ~51 kb. BUSCO assessment indicated 99.1% completeness (98.8% single-copy, 0.4% duplicated), with GC content at 44.8% and low gap content (0.053%).

### Genome annotation

Repeats annotation

Annotation of repetitive elements in the genomes of *B. burdigala*, *B. chloropharynx*, and *B. schalleri* revealed that transposable elements (TEs) comprise a substantial portion of each genome, with notable variation in both content and composition among species ( Table 2). Retroelements were a dominant repeat category, accounting for 11.3% in BBS, 11.4% in *B. burdigala*, and up to 15.9% in *B. chloropharynx.* Among these, LINEs were the most prevalent, particularly the L2/CR1/Rex and L1/CIN4 subfamilies, followed by LTR elements such as Gypsy/DIRS1 and BEL/Pao, which collectively contributed to the structural and regulatory diversification of the genome. Although SINEs and Penelope elements were detected, they represented only a minor fraction of the total retroelement content (<0.3%).

**Table 2.** Classification of repeat elements of *B. burdigala*, *B. chloropharynx*, and *B. schalleri* genome assemblies.

Repeat category	Percentage of sequence (%)		
	*Betta burdigala*	*Betta chloropharynx*	*Betta schalleri*
Retroelements	**5.98**	**8.39**	**6.18**
SINEs	0.21	0.42	0.39
Penelope	0.03	0.01	0.32
LINEs	4.03	5.24	3.53
CRE/SLACS	0	0	0
L2/CR1/Rex	2.53	3.31	2.22
R1/LOA/Jockey	0.17	0.24	0.11
R2/R4/NeSL	0.25	0.37	0.36
RTE/Bov-B	0.41	0.8	0.54
L1/CIN4	0.58	0.35	0.16
BEL/Pao	0.11	0.25	0.21
Ty1/Copia	0.01	0.02	0.03
Gypsy/DIRS1	0.97	1.53	1.14
Retroviral	0.43	0.65	0.32
DNA transposons	**3.85**	**5.54**	**4.02**
hobo-Activator	1.18	1.86	1.5
Tc1-IS630-Pogo	0.68	1.65	1.08
En-Spm	0	0	0
MULE-MuDR	0	0	0
PiggyBac	0.13	0.2	0.1
Tourist/Harbinger	0.62	0.53	0.32
Rolling-circles	**0.04**	**0.19**	**0.03**
Unclassified	**4.21**	**5.93**	**3.94**
Total Interspersed	**14.03**	**19.86**	**14.14**
Small RNA	**0.12**	**0.34**	**0.25**
Satellites	**0.06**	**0.07**	**0.05**
Simple repeats	**2.14**	**1.59**	**1.82**
Low complexity	**0.23**	**0.21**	**0.22**
Total Masked Bases	**16.54**	**22.08**	**16.30**

DNA transposons were the second most abundant repeat class, comprising 6.5%, 9.8%, and 7.0% of the genomes of *B. burdigala*, *B. chloropharynx*, and *B. schalleri*, respectively ( Table 2). The dominant families within this group included hobo-Activator, Tc1-IS630-Pogo, and En-Spm elements. These transposons are known for their cut-and-paste mechanism, and their relative abundance suggests that DNA-mediated transposition has played an important role in shaping genome architecture, particularly in BBC where their proportion was the highest. Interestingly, MULE-MuDR and PiggyBac elements were detected at low levels or were absent, suggesting potential lineage-specific losses or underrepresentation due to assembly or annotation limitations.

In addition to classified repeat categories, a significant fraction of the genome (3.9–5.9%) consisted of unclassified elements, indicating the presence of either novel transposable elements or highly diverged copies that defy current classification models ( Table 2). Moreover, 22–30% of repetitive content across the three genomes fell into the “Other” category, representing repeat families that could not be assigned confidently to standard classifications. The higher repeat content observed in *B. chloropharynx*, particularly in retrotransposons and unclassified repeats, may reflect recent TE amplification events or reduced efficacy of TE suppression mechanisms in this lineage.

Structural and functional annotation and gene duplication analysis

The genome annotation summary across the three Betta species— *B. burdigala*, *B. chloropharynx*, and *B. schalleri*—revealed a broadly similar genomic architecture, with minor differences in gene structure metrics ( Table 3). All three species exhibit approximately 1.3 transcripts per gene on average, and the number of predicted transcripts ranged from 31,517 in *B. burdigala* to 32,786 in *B. chloropharynx.* The mean exon size was consistent (172–174 bp), while the mean gene locus size ranged from 8,784 bp in *B. burdigala* to 9,013 bp in *B. chloropharynx.* Notably, the percentage of genes with alternative transcript variants was relatively stable across species (~23%), and most genes were multi-exonic (over 91%), indicating conserved splicing complexity in the genus.

**Table 3.** Genome annotation summary for the genome assembly of *Betta burdigala*, *Betta chloropharynx*, and *Betta schalleri.*

Statistic	Betta burdigala (BBB)	Betta chloropharynx (BBC)	Betta schalleri (BBS)
Max transcripts/gene	12	11	11
Mean exon size (bp)	173.2	174.1	172.7
Mean gene locus size (bp)	8784.6	9013.1	8942.5
Mean distinct exons/gene	10.4	10.2	10.4
Mean transcripts/gene	1.3	1.3	1.3
Mean transcript size (bp)	2013.8	1995.4	2016.1
Number of distinct exons	245812	251941	247092
Number of genes	23645	24597	23767
Genes with alt. transcripts	5,440 (23.0%)	5,615 (22.8%)	5,479 (23.1%)
Multi-exon genes	21,835 (92.3%)	22,592 (91.8%)	21,922 (92.2%)
Predicted transcripts	31517	32786	31694
Single-exon genes	1,810 (7.7%)	2,005 (8.2%)	1,845 (7.8%)

**Figure 4.**
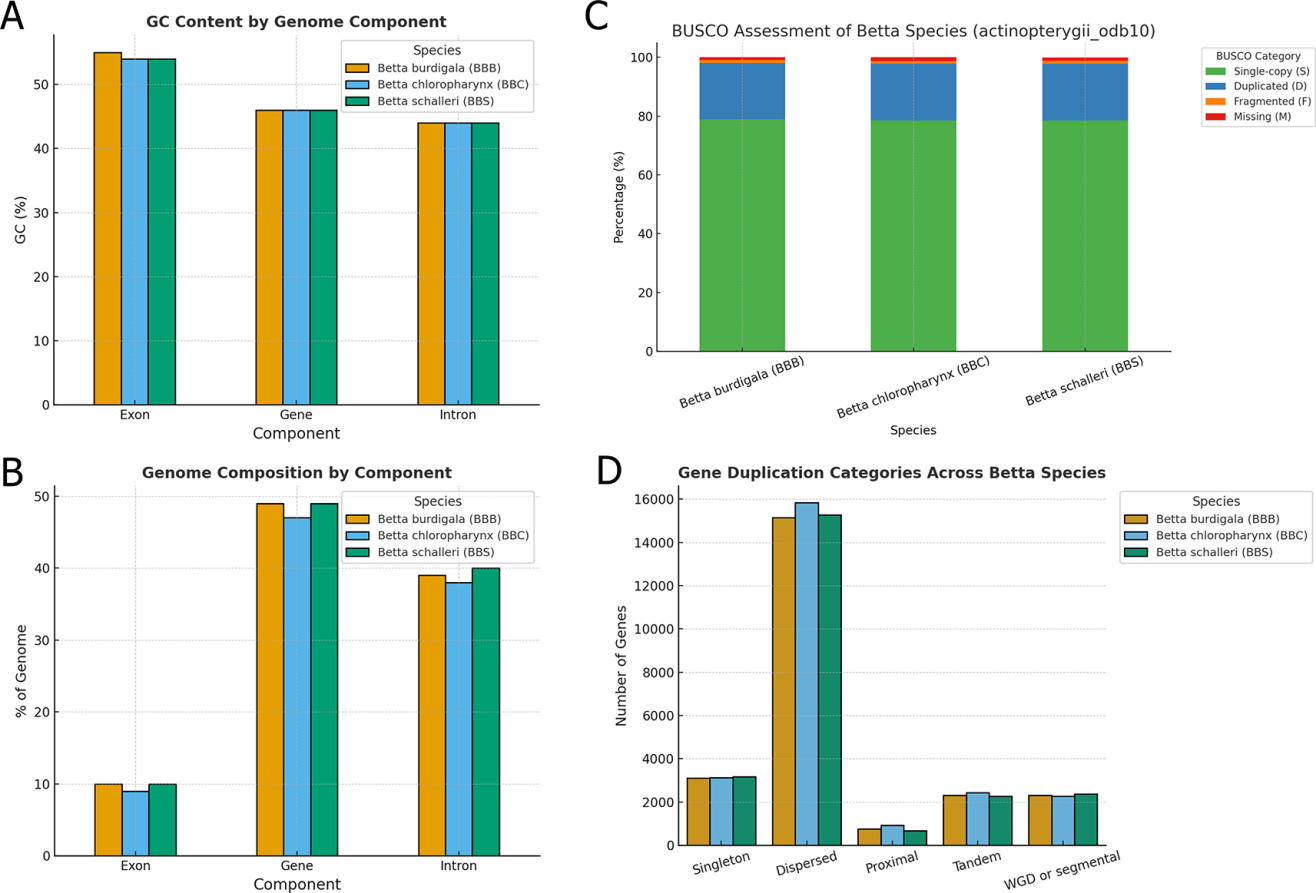
Genome annotation statistics and assessment of *Betta burdigala*, *Betta chloropharynx*, and *Betta schalleri.* (A) GC content by component of the three betta species genome assemblies. (B) Genome composition by component of the three Betta species genome assemblies. (C) BUSCO assessment of genome annotation results for the three Betta species genome assemblies. (D) Gene duplication classification in the genome of the three Betta species. Note that a gene can be classified into more than one gene duplication type.

Regarding genome composition, the GC content of genes and introns was consistent across species at 46% and 44%, respectively ( Figure 4A). Exonic GC content was slightly higher, ranging from 54% in *B. chloropharynx* and *B. schalleri* to 55% in *B. burdigala* ( Figure 4B). Exons accounted for 9–10% of the genome, while genes (including exons and introns) occupied approximately 47–49% ( Figure 4B). Introns alone contributed 38–40% of the genome, reflecting their substantial role in genome size and structure ( Figure 4B). These values suggest that, despite minor quantitative differences, the overall organization and composition of the genomes are highly conserved across the Betta species analyzed.

The BUSCO analysis using the *actinopterygii_odb10* dataset revealed high completeness across all three *Betta* species proteomes, indicating high-quality gene annotations ( Figure 4C). *Betta burdigala* showed the highest completeness with 98.0% of BUSCOs identified as complete, closely followed by *B. schalleri* at 97.9%, and *B. chloropharynx* at 97.8%. The majority of these complete BUSCOs were single-copy, representing 78.5–78.8% of the total, while duplicated BUSCOs accounted for approximately 19.2–19.3%. This elevated duplication level likely reflects the inclusion of multiple transcript isoforms in the complete predicted protein sets rather than extensive true biological duplication. Nevertheless, the high proportion of complete BUSCOs supports the overall completeness of the genome annotations.

We also explored the gene duplication patterns revealing notable similarities and subtle differences in the distribution of duplication types among the three *Betta* species ( Figure 4D). Across all species, dispersed duplications were the most prevalent, with *B. chloropharynx* showing the highest count (15,831 genes), followed closely by *B. schalleri* (15,269 genes) and *B. burdigala* (15,157 genes). This suggests that dispersed duplications represent a dominant mechanism contributing to gene expansion in these genomes, likely reflecting ongoing evolutionary pressures and functional diversification.

Other duplication categories, including tandem, proximal, and whole-genome duplication (WGD)/segmental events, were represented in roughly similar proportions across species ( Figure 6). Tandem duplicates ranged from 2,277 in *B. schalleri* to 2,437 in *B. chloropharynx*, while proximal duplications were more variable, with *B. schalleri* showing the lowest count (673) compared to 924 in *B. chloropharynx.* WGD or segmental duplicates remained relatively consistent across species (approximately 2,270–2,370), highlighting a shared genomic history of large-scale duplication events. Singleton genes, which lack detectable paralogs, accounted for 3,114–3,178 genes depending on the species. The identified duplication patterns should therefore be interpreted as comparative genomic patterns inferred from predicted gene models and homologous gene relationships, rather than as definitive evidence of fully functional duplicated genes.

### Comparative genomic

**Figure 5.**
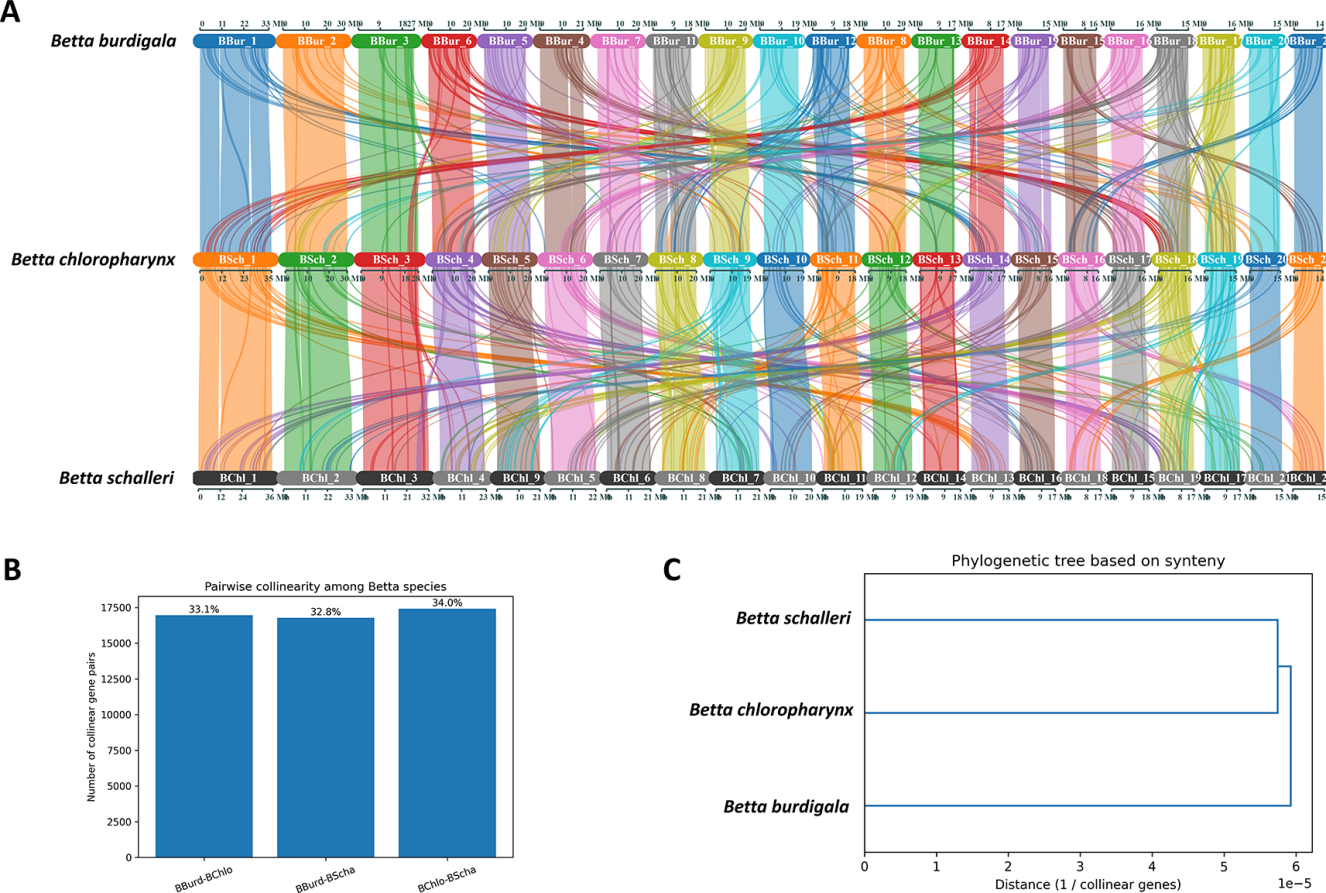
Collinearity analysis of genome assembly of *Betta burdigala* (BBur), *Betta schalleri* (BSch), and *Betta chloropharynx* (BChl). (A) syntenic block connection of the three genomes. (B) Pairwise collinearity among Betta species. (C) Pylogenetic tree based on synteny.

We conducted a collinearity analysis to investigate genome-level relationships among *B. burdigala*, *B. chloropharynx*, and *B. schalleri.* The analysis identified conserved syntenic gene blocks between each pair of species, which were subsequently quantified as collinear gene pairs ( Figure 5A). A total of 16,948 pairs were detected between *B. burdigala* and *B. chloropharynx* (33.15%), 16,778 pairs between *B. burdigala* and *B. schalleri* (32.81%), and 17,405 pairs between *B. chloropharynx* and *B. schalleri* (34.04%). These values indicate a broadly conserved syntenic patterns across all three species, with each pair retaining roughly one-third of the total detected collinear relationships.

Among the three comparisons, the highest level of collinearity was observed between *B. chloropharynx* and *B. schalleri*, suggesting that these two species are slightly more closely related to each other than to *B. burdigala* ( Figure 5B). In contrast, *B. burdigala* shows a similar but marginally lower level of collinearity with both *B. chloropharynx* and *B. schalleri*, implying that *B. burdigala* likely diverged earlier from the lineage leading to *B. chloropharynx* and *B. schalleri.* Visualization of the results using a dendrogram and a network graph further supports this interpretation. The dendrogram places *B. chloropharynx* and *B. schalleri* as sister species, with *B. burdigala* forming an outgroup ( Figure 5C). The network graph illustrates conserved syntenic connections among all three species, but with the thickest edge linking *B. chloropharynx* and *B. schalleri*, reflecting their higher degree of shared genome organization.

**Figure 6.**
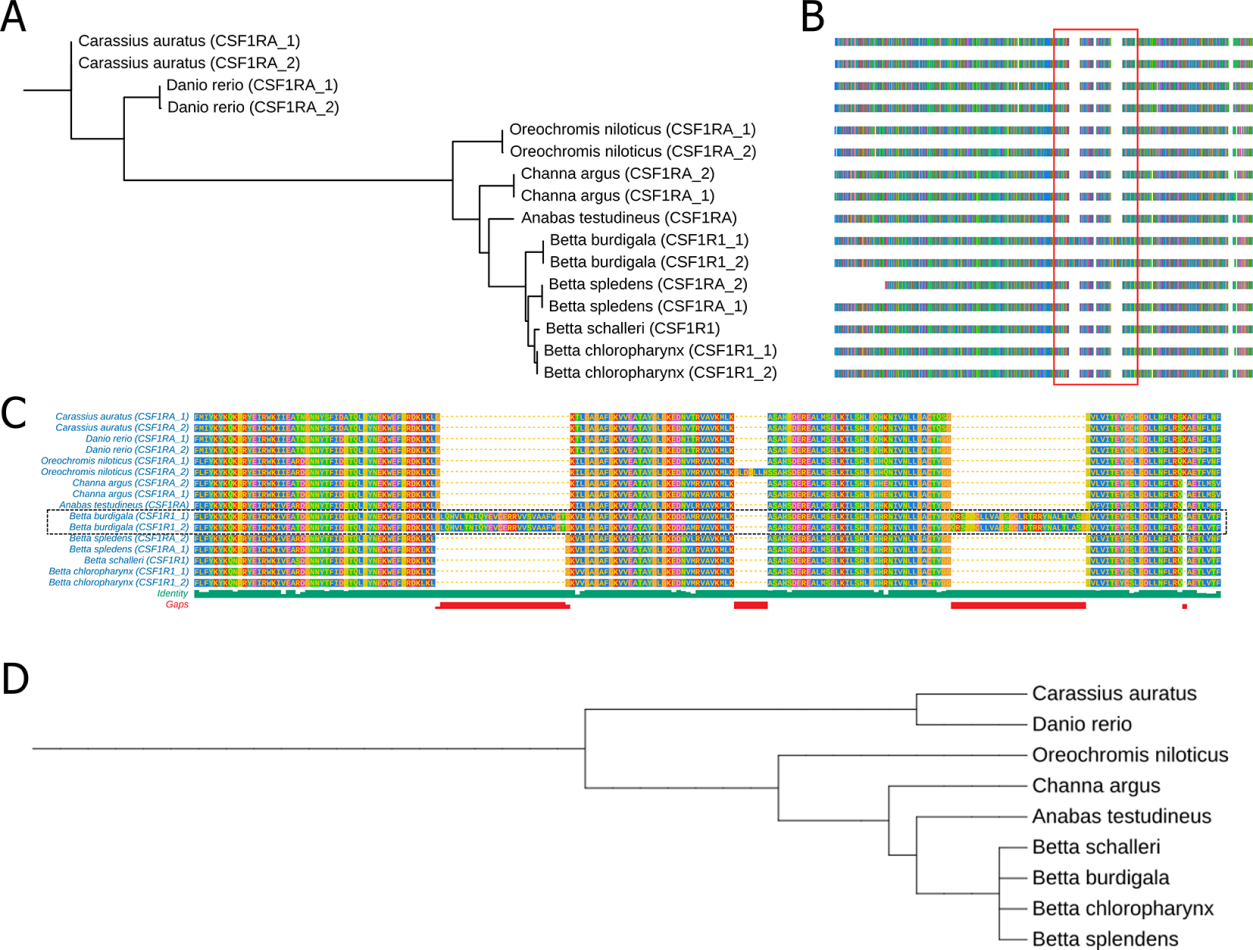
A comparative analysis of the Colony Stimulating Factor 1 Receptor A (CSF1RA) protein sequence among *B. burdigala*, *B. chloropharynx*, and *B. schalleri*, alongside *B. splendens*, *Anabas testudineus*, *Channa argus, Oreochromis niloticus, Danio rerio, and Carassius auratus.* (A) A phylogenetic tree derived from CSFRA protein alignments across nine fish species. (B) Multiple Sequence Alignment of the CSFRA protein in these nine fish species. (C) A zoomed-in view of the sequence alignment within the red line box in (B). (D) a phylogenetic tree of nine fish species based on NCBI taxonomic data. The complete protein sequence alignment of CSF1RA is available in Supplementary file 1.

We also did comparative analysis of three genes that might involve in color patterning of fishes which are Colony Stimulating Factor 1 Receptor A (CSF1RA), Melanocortin 1 Receptor (MC1R) and Paired Box 7 (PAX7). The protein sequence alignment are available in supplementary files (Supplementary file 1, Supplementary file 2, and Supplementary file 3). Gene-based phylogenetic analyses showed that the CSF1RA tree exhibited clustering patterns broadly consistent with the established taxonomic relationships among the nine fish species based on NCBI taxonomy ( Figure 6A and D). We note that these analyses are based on single genes and therefore are intended primarily for comparative assessment of gene-level evolutionary patterns rather than for robust species-level phylogenetic inference. On the other hand, we observed that the branching pattern of the MC1R and PAX7 phylogenetic tree does not align with the species phylogenetic tree (Suppl Fig 2). We also identified two significant protein insertions in the CSF1RA of *B. burdigala* ( Figure 6B and C). The first insertion, consisting of 27 amino acids, is located at approximately alignment position 594, just before the start of the Kinase Domain (GKVLGAGAFG…). The second insertion, comprising 28 amino acids, is situated at around alignment position 701, between the two lobes of the kinase domain.

## Discussion

Our study integrates meristic analyses and genome assemblies to provide a comparative framework for understanding divergence among three endemic *Betta* species from Bangka Island: *B. burdigala*, *B. chloropharynx*, and *B. schalleri.* Morphological comparisons revealed clear diagnostic traits that distinguish these species and align with their current taxonomic placement. *B. burdigala* showed reductions in dorsal and predorsal scale counts, consistent with its placement in the *coccina* group. ^33^ *B. chloropharynx* was distinguished by its characteristic throat markings and opercular coloration typical of the *waseri* group, ^35^ while *B. schalleri* exhibited greater fin ray counts and deeper body proportions, traits associated with the *pugnax* group. ^36^ These meristic patterns reinforce species boundaries and highlight morphological divergence even within closely related taxa.

The genomic assemblies presented here represent the first references for these Bangka endemics. All three species demonstrated high contiguity and completeness, confirming their reliability for downstream analyses. Subtle genomic differences were also evident. *B. chloropharynx* displayed the largest genome size, highest scaffold N50, and elevated retrotransposon content, suggesting lineage-specific repeat dynamics. Gene duplication profiles showed dispersed duplications as the dominant category across all species, while variation in tandem and proximal duplications may indicate lineage-specific expansions. ^37– 39^

These findings demonstrate that while overall genome organization and gene content remain conserved across the three species, lineage-specific signatures of divergence are detectable. The combination of high collinearity and subtle differences in repeat content and duplication patterns provides a genomic basis for understanding evolutionary relationships within the genus. ^40, 41^ Moreover, the integration of meristic and genomic evidence strengthens taxonomic resolution, particularly in lineages where morphology alone may be insufficient.

Together, the annotation and composition data indicate a strong conservation of gene architecture and genomic content within the *Betta* species, consistent with their relatively recent divergence. Subtle differences, such as the slightly higher transcript count or gene locus size in *B. chloropharynx*, may hint lineage-specific regulatory or structural variations. ^42, 43^ The consistent duplication profiles across the three species suggest conserved duplication dynamics within the *Betta* genus. However, the modest variation in tandem and proximal duplication counts may point to lineage-specific gene family expansions or contraction events, potentially linked to evolutionary divergence among these lineages. ^38, 44^ However, population-level genomic data will be required to definitively connect these structural variations to specific ecological adaptations.

Collinearity analysis revealed broadly conserved syntenic patterns among the three *Betta* species, with *B. chloropharynx* and *B. schalleri* showing comparatively higher levels of shared gene order and synteny relative to *B. burdigala*. These patterns are generally consistent with their closer evolutionary relationship inferred from existing taxonomic and comparative genomic evidence. Overall, the results suggest that the three *Betta* species retain largely conserved genome organization, while *B. chloropharynx* and *B. schalleri* may share more similar genomic architectures compared to *B. burdigala*. Because the assemblies were scaffolded using a reference-guided approach, these collinearity patterns should be interpreted cautiously as indicators of broad-scale syntenic conservation rather than definitive evidence of fully conserved chromosomal structure. Nevertheless, the analyses provide a useful comparative genomic framework for exploring genome organization and evolutionary relationships within the *Betta* species. ^42, 45, 46^ The observed syntenic conservation, particularly between *B. chloropharynx* and *B. schalleri*, is also consistent with patterns reported in other closely related fish taxa, where substantial conservation of gene order is often retained despite lineage-specific chromosomal rearrangements in more divergent groups. ^47, 48^

Our comparative genomic analysis reveals a two-layered evolutionary dynamic underlying pigmentation diversity in Betta species: strong conservation of core pigment-cell survival pathways alongside rapid divergence in genes governing color type and pattern formation. CSF1RA, a key regulator of melanophore and color pigments (xanthophores, erythrophores, and iridophore) development, ^49^ ^–^ ^51^ exhibited clustering patterns broadly consistent with the accepted species relationships and shows high sequence conservation across all examined fishes, reflecting high sequence conservation and potential evolutionary constraints on this essential signaling pathway. Notably, however, *B. burdigala* exhibits two large amino-acid insertions positioned adjacent to and within the kinase domain, indicating species-specific modulation rather than disruption of receptor function. While further research is necessary, these insertions may contribute to modulation of pigment patterning or coloration intensity, potentially enhancing this species’ vibrant coloration. In contrast, genes associated with pigment-cell fate and color intensity—particularly PAX7 and MC1R— showed topologies that differed from the accepted species relationships. Divergence in PAX7, a major regulator of xanthophore and erythrophore lineages, ^52^ ^–^ ^54^ is consistent with adaptive shifts affecting red–yellow pigmentation, especially relevant given the pronounced red coloration in *B. burdigala.* Similarly, the non-concordant evolution of MC1R, despite its variable role in teleost pigmentation, ^55^ ^–^ ^57^ 4 is consistent with high sequence divergence that may warrant future investigation into potential regulatory or functional divergence within the *Betta* genus. Together, these findings indicate that *Betta* color diversity arises from conserved developmental frameworks overlaid by lineage-specific sequence variation and structural innovation, enabling fine-scale modulation of pigment cell types and color intensity across species. ^58^

Although our data do not directly address ecological or paleogeographic processes, previous studies suggest that the high endemism of Bangka’s *Betta* species has been shaped by Pleistocene paleogeography and the persistence of specialized peat swamp habitats. ^6, 59– 64^ In this context, the divergence patterns we observed in both morphology and genomics are consistent with scenarios of historical geographic isolation and ecological filtering proposed by earlier work. Our results therefore provide the genetic and morphological evidence that complements these broader biogeographic hypotheses. We acknowledge that the genome assemblies generated in this study are based on single individuals for each species and therefore may not fully represent the extent of intraspecific genomic variation present within natural populations. Nevertheless, these genomic resources contribute important data for species delimitation and phylogenomic placement within the *Betta* genus, while also establishing a foundation for future studies investigating the relationships among genomic variation, ecology, adaptation, and conservation. Given the conservation status of these taxa, the genomic resources generated here are valuable for monitoring genetic diversity and informing management strategies for Bangka’s unique freshwater biodiversity.

## Conclusion

This study provides the first integrated meristic and genomic comparison of three endemic Betta species from Bangka Island— *B. burdigala*, *B. chloropharynx*, and *B. schalleri.* Meristic analyses confirmed diagnostic traits supporting their taxonomic distinction, while high-quality genome assemblies with near-complete BUSCO scores (>97%) offer reliable references for evolutionary and conservation studies. Although overall genomic architecture is conserved, subtle differences in genome size, repeat composition, and duplication profiles highlight lineage-specific divergence, with synteny analyses indicating a closer relationship between *B. chloropharynx* and *B. schalleri.* By combining morphological and genomic evidence, this study not only strengthens the resolution of species boundaries but also establishes essential resources for monitoring genetic diversity and informing conservation strategies for these endangered freshwater fishes.

## Data Availability

The data related to this project have been deposited under NCBI BioProject Accession PRJNA1328177 and are publicly available. The biosample accession numbers are SAMN51307167, SAMN51307168, and SAMN51307169 for *B. burdigala*, *B. schalleri*, and *B. chloropharynx* respectively. The raw genome sequence reads have been deposited in the NCBI Sequence Read Archive (SRA) under the accession numbers SRR35652347 for *B. burdigala*, SRR35653255 for *B. schalleri*, and SRR35728893 and SRR35728892 for *B. chloropharynx.* Additionally, the genome assemblies have been deposited in the NCBI GenBank database with accession numbers GCA_054471155.1, GCA_054471145.1, GCA_054471135.1 for *B. burdigala*, *B. schalleri*, and *B. chloropharynx*, respectively. The genome assemblies and their annotations have also been deposited and are available in the National Scientific Repository of the National Research and Innovation Agency (Badan Riset dan Inovasi Nasional/BRIN) of the Republic of Indonesia: Genome Betta spp. ( https://hdl.handle.net/20.500.12690/RIN/LEOQPP). ^65^ All extended data have been deposited in figshare (DOI: https://doi.org/10.6084/m9.figshare.31189546.v3). ^66^ The extended data include the following: Suppl Fig 1. The meristic (A) and morphometric (B) characters of *Betta spp.* Suppl Fig 2. A comparative analysis of the Melanocortin 1 Receptor (MC1R) and Paired box 7 (PAX7) protein sequences among *B. burdigala*, *B. chloropharynx*, and *B. schalleri*, alongside *B. splendens, Anabas testudineus, Channa argus, Oreochromis niloticus, Danio rerio, and Carassius auratus.* (A) A phylogenetic tree derived from MC1R protein alignments across nine fish species. (B) Multiple Sequence Alignment of the PAX7 MC1R protein in these nine fish species. (C) A phylogenetic tree derived from PAX7 protein alignments across nine fish species. (D) Multiple Sequence Alignment of the PAX7 protein in these nine fish species. The complete protein sequence alignment of MC1R and PAX7 are available in Supplementary file 2 and Supplementary file 3 respectively. Suppl Fig 3. Validation of the CSF1R1 insertion using Oxford Nanopore long-read alignments. Coverage depth across the insertion locus remained consistent (~70–88×), and multiple long reads spanned the insertion region continuously, supporting the authenticity of the predicted insertion and excluding local assembly artifacts. Supplementary file 1. Protein alignment of CSF1R1 in 9 fishes.clustal_num Supplementary file 2. Protein alignment of MC1R in 9 fishes.clustal_num Supplementary file 3. Protein alignment of PAX7 in 9 fishes.clustal_num Supplementary file 4. Coverage depth of CSF1R1_1 region by Oxford Nanopore long-read alignments. Supplementary file 5. QUAST assembly quality assessment statistics for the three *Betta* genome assemblies. The complete checklists of ARRIVE 2.0 reporting guidelines is available at https://doi.org/10.6084/m9.figshare.31136455. ^10^ All the underlying and extended data of this study are openly available under the terms of The CC BY 4.0 license (Creative Commons Attribution 4.0 International).
