# Supplementary material for: Beyond the Beauty: Meristic and Genomic Signatures of Bangka’s Endemic Betta Fishes
Source: F1000Res. 2026 Jun 1;15:226. Originally published 2026 Feb 10. [Version 2] doi: 10.12688/f1000research.174779.2 (PMC13254521; doi:10.12688/f1000research.174779.2)
Supplement: Peer review history

# Reviewer report: Reviewer response for version 2

**DOI:** 10.5256/f1000research.202000.r489884

**Published:** 2026-06-13

**Authors:** Bingpeng Xing

**Affiliations:** 1 Third Institute of Oceanography Ministry of Natural Resources, Xiamen, China

The authors have adequately addressed the concerns raised during the previous round of review. In particular, they have improved methodological transparency, added genome quality metrics and contamination screening, clarified the limitations of reference-guided scaffolding and single-gene phylogenetic analyses, and provided additional evidence supporting the reported CSF1RA insertions. The remaining issues are mainly minor language and copyediting problems and do not affect the validity of the data or the conclusions. I believe the manuscript is now suitable for indexing and recommend approval.

Is the work clearly and accurately presented and does it cite the current literature?

Partly

If applicable, is the statistical analysis and its interpretation appropriate?

Partly

Are all the source data underlying the results available to ensure full reproducibility?

Yes

Is the study design appropriate and is the work technically sound?

Partly

Are the conclusions drawn adequately supported by the results?

Partly

Are sufficient details of methods and analysis provided to allow replication by others?

Partly

Reviewer Expertise:

marine biology，genome，fish，genetic，DNA，eDNA

I confirm that I have read this submission and believe that I have an appropriate level of expertise to confirm that it is of an acceptable scientific standard.

# Reviewer report: Reviewer response for version 2

**DOI:** 10.5256/f1000research.202000.r491035

**Published:** 2026-06-10

**Authors:** Axel Janke

**Affiliations:** 1 Senckenberg Biodiversity and Climate Research Centre, Frankfurt am Main, Germany

The genome data are interesting and clearly worth publishing. The authors present a high-quality assembly of yet another species in the genus, supported by standard descriptive analyses, clear graphics, and some intra-genus comparisons. Overall, the technical work is solid and well executed.

 However, I find the biological and scientific novelty rather limited. While the genome itself is a useful addition, I am not particularly convinced by the single-gene phylogenetic analyses presented, especially given that the full genome is available. More comprehensive genome-wide analyses would have strengthened the study significantly.

  If possible, cite the first Betta genome: (Ref 1 - Prost S, et al., 2020) Prost S, Petersen M, Grethlein M, et al. Improving the Chromosome-Level Genome Assembly of the Siamese Fighting Fish ( *Betta splendens*) in a University Master's Course. G3 (Bethesda, Md.). 2020 Jul;10(7):2179-2183. DOI: 10.1534/g3.120.401205. PMID: 32385046; PMCID: PMC7341155.

Is the work clearly and accurately presented and does it cite the current literature?

Yes

If applicable, is the statistical analysis and its interpretation appropriate?

Yes

Are all the source data underlying the results available to ensure full reproducibility?

Yes

Is the study design appropriate and is the work technically sound?

Yes

Are the conclusions drawn adequately supported by the results?

Yes

Are sufficient details of methods and analysis provided to allow replication by others?

Yes

Reviewer Expertise:

Mammalian evolution, population genomics, giraffe, bear, whales, wolves, wolverines, genome assembly

I confirm that I have read this submission and believe that I have an appropriate level of expertise to confirm that it is of an acceptable scientific standard.

# Reviewer report: Reviewer response for version 1

**DOI:** 10.5256/f1000research.192703.r457789

**Published:** 2026-03-05

**Authors:** Nicholas S Kron

**Affiliations:** 1 University of Miami, Rickenbacker Cswy, Miami, USA; 2 Genevia Technologies, Finland, Finland

**Summary**

 In “Beyond the Beauty: Meristic and Genomic Signatures of Bangka’s Endemic Betta Fishes”, Helmizuryani et al. describe the collection, sampling, genome assembly and annotation, and comparative genomic analysis of three species of the genus *Betta* from the Bangka islands of Indonesia. The authors demonstrate high quality, sub-chromosome level genome assemblies using Oxford Nanopore sequencing and established long read assembly and annotation workflows. Based on genome annotation results, the authors describe the genomes of the three species as highly colinear and consistent, with minor variation among them possibly relating to evolutionary divergence among the three, with special attention paid to pigmentation associated genes.   

**Comments**

 The study employs commonly used open-source tools and standard methodologies for assembly and annotation. The genomic resources generated by this study are novel and valuable contributions and will surely be useful for future studies in these species and for teleost and vertebrate genomics more broadly.  

 The manuscript would benefit from proof reading for spelling, grammar, consistency, etc.:

- Consistency of italicization for species names throughout.
- Introduction: “The number of endemic wild Betta distribution and diversity of Southeast Asia has been identified for 16 years in Thailand (2 species) and Indonesia (9 species)”
- Introduction: “Three species of Betta spp. were endemic, i.e., *Betta burdigala*, *Betta chloropharynx*, and *Betta schalleri.”*
- Introduction: “The genetic studies of the endemic fish Betta spp. on Bangka Island have…”
- Introduction: “However, the whole genome for the evolution of the endemic fish Betta spp. on Bangka Island hasn’t been revealed.”
- Introduction: “High-quality genomic data could be used for Long-read sequencing and high-throughput chromosome (Hi-C) technology.”
- The authors reference the benefits of Hi-C in their introduction but do not use Hi-C in their analysis.

**Are sufficient details of methods and analysis provided to allow replication by others? (partly)**

 The authors provide the software and version numbers to software used which is appreciated and facilitates reproducibility. However, the authors do not specify if software was used with default parameters or parameters deviating from default. These can be critical for reproducing or evaluating the analyses done, especially for tools like BLAST. Nor do the authors provide links to repositories (e.g. github, zenodo) of scripts used for verification and/or reproduction of methods. I would strongly recommend a remark in the methods stating that unless otherwise specified, default parameters are used, and explicit description when non-standard parameters are used in text or a supplement. If possible, provide access to analysis scripts in their entirety.

**Are the conclusions drawn adequately supported by the results? (Partly)**

1. The assembly BUSCO assessments have a very low duplicate BUSCO count but the gene model level BUSCO assessments are much higher (~20%). This is not unexpected as isoform redundancy, pseudo-genes, etc do crop up and can impact BUSCO analysis. Have the authors run the annotation level BUSCO analysis using only longest isoforms to verify? It is not clear from the methods how exactly BUSCO was run on the annotation results.
2. The authors do not mention any secondary verification on the gene models / predicted duplicates to verify they are not artifacts/pseudogenes/etc. While automated pipelines like funannotate are powerful and convenient, secondary validation of gene models is often needed especially if proceeding to duplication analysis with tools like MCScanX/OrthoFinder/Café.
3. The authors do not describe the assembly or annotation of the mitochondrial genome of each species. While not strictly necessary per se, these data are readily isolated from the long reads and would provide a more complete picture of the genomes and further context to the syntenty-based phylogenetic analysis.
4. The authors use RagTag for reference-based scaffolding of assembled contigs. In the absence of Hi-C/OmniC data, this is a suitable alternative, especially given the authors have access to a closely related congener *Betta splendens*. This success of this approach is determined by the completeness/continuity/scaffold size of the input assemblies and the reference. Given this, comparisons of the assembly and annotation statistics of the new assemblies to the reference would provide important context for these assemblies. This would improve the ability of readers to gauge the quality of the assemblies and the success of the method. For example, a table produced by a tool like QUAST that compares the major assembly statistics would be suitable.
5. The RagTag method used can cause scaffolded assemblies to resemble the reference genome in terms of structure, especially for more fragmented/low coverage assemblies. The authors need to clearly discuss the potential impact of these potential artefacts on their collinearity results and conclusions.
6. Given the authors are using gene models for their comparative analysis (not sequences directly verified with molecular methods), the authors should report support for identified protein insertions with primary data (e.g. insertion spanning long reads, depth of coverage at the insertion site, etc.).   
7. In the discussion, the authors make claims about purifying selection, rate of gene evolution, lineage specific selection, etc.. These are inferences that can only be drawn with population genetic data. These claims are improper for the data presented (e.g. assemblies of single individuals) and should be removed or qualified as such.

**Additional comments**

1. The manuscript would be strengthened by reporting metrics from initial k-mer profiling with GenomeScope2. These could be reported in the form of the annotated k-mer distribution plot generated by GenomeScope2.
2. The authors do not describe any methods for decontamination (either technical or biological). If these were not performed, this should be explicitly noted. If they were, what methods were employed and if they were pre-assembly or post assembly should be specified.
3. The authors do make publicly available the raw data, genome assemblies, and annotations. I would also recommend additionally submitting the repeats to public databases such as Dfam or FishTEDB.

Is the work clearly and accurately presented and does it cite the current literature?

Yes

If applicable, is the statistical analysis and its interpretation appropriate?

I cannot comment. A qualified statistician is required.

Are all the source data underlying the results available to ensure full reproducibility?

Yes

Is the study design appropriate and is the work technically sound?

Yes

Are the conclusions drawn adequately supported by the results?

Partly

Are sufficient details of methods and analysis provided to allow replication by others?

Partly

Reviewer Expertise:

bioinformatics, marine biology

I confirm that I have read this submission and believe that I have an appropriate level of expertise to confirm that it is of an acceptable scientific standard, however I have significant reservations, as outlined above.

# Response

**Published:** 2026-05-18

**Authors:** Luthfi Nurhidayat

**Affiliations:** Faculty of Biology Universitas Gadjah Mada, Indonesia

**Comments**

 The study employs commonly used open-source tools and standard methodologies for assembly and annotation. The genomic resources generated by this study are novel and valuable contributions and will surely be useful for future studies in these species and for teleost and vertebrate genomics more broadly.  

 The manuscript would benefit from proof reading for spelling, grammar, consistency, etc.:

- Consistency of italicization for species names throughout.

**Response:**

 We have checked and fixed the consistency of italicization for species names within the revised manuscript.

  

- Introduction: “The number of endemic wild Betta distribution and diversity of Southeast Asia has been identified for 16 years in Thailand (2 species) and Indonesia (9 species)”

**Response:**

 We have revised it to “Indonesia harbors nine wild Betta species classified as Critically Endangered (CN) and seven species categorized as Endangered (EN). In comparison, Thailand contains two Critically Endangered (CE) wild Betta species and one Endangered (EN) species”

  

- Introduction: “Three species of Betta spp. were endemic, i.e., *Betta burdigala*, *Betta chloropharynx*, and *Betta schalleri.”*

**Response:**

 We have revised it to “Three *Betta* species— *B. burdigala*, *B. chloropharynx*, and *B. schalleri*—are endemic to the Bangka Islands”

- Introduction: “The genetic studies of the endemic fish Betta spp. on Bangka Island have…”

**Response:**

 We have change it to “Previous studies on the endemic *Betta* species from Bangka Island have included genetic analyses based on mitochondrial DNA and eDNA metabarcoding, as well as comprehensive morphological examinations, all of which have contributed to clarifying the genetic relationships and taxonomic status of these species.”

- Introduction: “However, the whole genome for the evolution of the endemic fish Betta spp. on Bangka Island hasn’t been revealed.”

**Response:**

 We have revised it to “However, the whole-genome evolutionary landscape of the endemic *Betta* species from Bangka Island has not yet been explored.”

- Introduction: “High-quality genomic data could be used for Long-read sequencing and high-throughput chromosome (Hi-C) technology.”

**Response:**

 Thank you for noticing the mistakes.  We have revised it to “High-quality genomic data can be generated using long-read sequencing technologies such as Oxford Nanopore sequencing, which enables the production of highly contiguous genome assemblies.....”

- The authors reference the benefits of Hi-C in their introduction but do not use Hi-C in their analysis.

**Response:**

 We have revised the Introduction section by removing the Hi-C-related description to better align the manuscript with the actual sequencing and assembly strategy used in this study, which was based on Oxford Nanopore long-read sequencing without Hi-C data. The revised text now reads:

 “High-quality genomic data can be generated using long-read sequencing technologies such as Oxford Nanopore sequencing, which enables the production of highly contiguous genome assemblies. These technologies help resolve complex repetitive regions and haplotype heterozygosity, both of which are major sources of genome assembly errors ^7^.”

 This revision ensures that the Introduction more accurately reflects the methodologies and analyses performed in the present study.

**Are sufficient details of methods and analysis provided to allow replication by others? (partly)**

 The authors provide the software and version numbers to software used which is appreciated and facilitates reproducibility. However, the authors do not specify if software was used with default parameters or parameters deviating from default. These can be critical for reproducing or evaluating the analyses done, especially for tools like BLAST. Nor do the authors provide links to repositories (e.g. github, zenodo) of scripts used for verification and/or reproduction of methods. I would strongly recommend a remark in the methods stating that unless otherwise specified, default parameters are used, and explicit description when non-standard parameters are used in text or a supplement. If possible, provide access to analysis scripts in their entirety.

**Response:**

 Thank you for this valuable suggestion. We agree that explicit reporting of software parameters and analytical workflows is essential for ensuring reproducibility and transparency. In response, we have revised the Methods section to clarify that, unless otherwise specified, all software tools were executed using their default parameters. We also added explicit descriptions for analyses in which non-default settings or filtering criteria were applied.

 Specifically, we added the following sentence to clarify the BLAST settings used in the duplication analysis:

 “The analyses were performed using an E-value threshold of 1e−3, with results limited to a maximum of five target hits per query sequence and output generated in tabular format (outfmt 6). Computations were executed using 12 parallel threads, while all other parameters were retained at their default settings.”

 Regarding the analysis scripts, most workflows in this study were conducted using established bioinformatics software through standard command-line execution rather than extensive custom scripting. Therefore, we focused on providing detailed descriptions of the analytical workflows, software versions, and key command parameters directly within the manuscript and supplementary materials to facilitate reproducibility. We also ensured that all non-default settings and critical filtering parameters are now explicitly documented in the revised manuscript.

 We appreciate the reviewer’s recommendation, which helped improve the methodological clarity, transparency, and reproducibility of the study.

**Are the conclusions drawn adequately supported by the results? (Partly)**

 1. The assembly BUSCO assessments have a very low duplicate BUSCO count but the gene model level BUSCO assessments are much higher (~20%). This is not unexpected as isoform redundancy, pseudo-genes, etc do crop up and can impact BUSCO analysis. Have the authors run the annotation level BUSCO analysis using only longest isoforms to verify? It is not clear from the methods how exactly BUSCO was run on the annotation results.

**Response:**

 Thank you for this important observation. We agree that annotation-level BUSCO assessments can show elevated duplicated BUSCO proportions due to the inclusion of multiple transcript isoforms, fragmented predictions, or potential pseudogene annotations.

 In our study, the BUSCO assessment of the predicted gene models was performed using the complete predicted protein set generated from the annotation pipeline, including all annotated isoforms, rather than restricting the analysis to only the longest isoform per gene. We acknowledge that this approach may contribute to the higher duplicated BUSCO proportion observed at the annotation level compared with the genome assembly-level BUSCO assessment.

 To clarify this point, we have revised the Methods section to explicitly describe how BUSCO was conducted on the annotation results, including the use of the full predicted protein dataset. We also added clarification in the Results that the elevated duplicated BUSCO values at the annotation level likely reflect transcript isoform redundancy and annotation complexity rather than extensive true biological duplication.

 Nevertheless, the overall high completeness of the predicted protein sets still supports the quality and comprehensiveness of the genome annotations generated in this study.

 2. The authors do not mention any secondary verification on the gene models / predicted duplicates to verify they are not artifacts/pseudogenes/etc. While automated pipelines like funannotate are powerful and convenient, secondary validation of gene models is often needed especially if proceeding to duplication analysis with tools like MCScanX/OrthoFinder/Café.

**Response:**

 Thank you for this important comment. We agree that automated genome annotation pipelines may occasionally retain fragmented gene models, pseudogenes, or assembly-related artifacts, and that additional validation can further improve confidence in downstream duplication analyses.

 In our study, structural gene prediction was performed using GALBA within the BRAKER framework. The GALBA pipeline incorporates protein evidence from closely related species to improve gene prediction accuracy and reduce false-positive annotations compared with purely ab initio approaches. Subsequently, Funannotate was used for functional annotation processing, including integration of functional annotation results into the GFF3 files and generation of submission-ready annotation files.

 The resulting annotations showed high completeness based on BUSCO assessment of the predicted protein sets, supporting the overall quality of the gene models used in downstream analyses. Consequently, the duplication analyses performed using MCScanX were based on curated predicted protein-coding genes generated from this evidence-supported annotation workflow.

 Nevertheless, we acknowledge that some predicted duplicates may still include fragmented annotations or non-functional copies that cannot be conclusively distinguished without additional transcriptomic, population-level, or experimental validation. To address this concern, we revised the manuscript in the “Structural and Functional Annotation and Gene Duplication Analysis” subsection of the Results section by adding the following statement:

 “The identified duplication patterns should therefore be interpreted as comparative genomic patterns inferred from predicted gene models and homologous gene relationships, rather than as definitive evidence of fully functional duplicated genes.”

 3. The authors do not describe the assembly or annotation of the mitochondrial genome of each species. While not strictly necessary per se, these data are readily isolated from the long reads and would provide a more complete picture of the genomes and further context to the syntenty-based phylogenetic analysis.

**Response:**

 Thank you for this insightful suggestion. We agree that mitochondrial genome assemblies can provide valuable complementary information for comparative and phylogenetic analyses. In the present study, however, our primary focus was on the nuclear genome assemblies and comparative analyses related to genome structure, synteny, and pigmentation-associated genes.

 We have in fact successfully assembled the mitochondrial genomes of the studied *Betta* species from the long-read sequencing data. However, these mitochondrial genome datasets are currently being analyzed in a separate study addressing distinct biological questions related to mitochondrial evolution and phylogeography. To avoid overlap between manuscripts and maintain a clear scope for the present study, we therefore chose not to include the mitochondrial genome analyses here.

 Nevertheless, we acknowledge the value of mitochondrial genomic information and have added a statement in the manuscript clarifying that mitochondrial genome assemblies were recovered from the sequencing data and will be reported separately in a dedicated study.

 4. The authors use RagTag for reference-based scaffolding of assembled contigs. In the absence of Hi-C/OmniC data, this is a suitable alternative, especially given the authors have access to a closely related congener *Betta splendens*. This success of this approach is determined by the completeness/continuity/scaffold size of the input assemblies and the reference. Given this, comparisons of the assembly and annotation statistics of the new assemblies to the reference would provide important context for these assemblies. This would improve the ability of readers to gauge the quality of the assemblies and the success of the method. For example, a table produced by a tool like QUAST that compares the major assembly statistics would be suitable.

**Response:**

 Thank you for this constructive suggestion. We agree that comparison of the newly generated assemblies against the reference genome provides important context for evaluating assembly quality and the effectiveness of the reference-guided scaffolding strategy.

 we performed additional assembly quality assessment using QUAST and have added the results as **Supplementary file 5. QUAST assembly quality assessment statistics for the three *Betta* genome assemblies **in figshare (DOI: https://doi.org/10.6084/m9.figshare.31189546.v3 ). The QUAST report includes major assembly statistics such as contig number, total assembly size, N50/NG50, largest contig size, GC content, duplication ratio, gap content, and misassembly statistics, together with alignment-based comparisons against the reference genome.

 The QUAST assessment showed that all three assemblies possess high contiguity, with contig N50 values ranging from ~19.3–20.1 Mb and largest contigs exceeding 33 Mb. In addition, the assemblies exhibited very low numbers of detected misassemblies and low gap content, supporting the overall structural quality of the assemblies.

 We believe the inclusion of the QUAST assessment substantially improves the contextual evaluation and transparency of the genome assemblies presented in this study.

 5. The RagTag method used can cause scaffolded assemblies to resemble the reference genome in terms of structure, especially for more fragmented/low coverage assemblies. The authors need to clearly discuss the potential impact of these potential artefacts on their collinearity results and conclusions.

**Response:**

 Thank you for this important comment. We acknowledge that the use of reference-guided scaffolding may influence large-scale structural organization and potentially increase apparent similarity to the reference genome. In our study, however, the genome assemblies were initially generated *de novo* from Oxford Nanopore long reads, while RagTag was used only during the scaffolding stage to improve chromosome-scale continuity.

 To address this concern, we have revised the Results and Discussion sections to more explicitly acknowledge the potential limitations associated with reference-guided scaffolding. We also clarified that the collinearity analyses were performed using annotated protein-coding genes and orthologous gene order on the scaffolded assemblies, rather than direct whole-genome nucleotide alignments. Therefore, the syntenic patterns presented in this study primarily reflect conservation of gene content and relative gene order, rather than exact chromosome-scale structural identity.

 In addition, we have moderated the wording throughout the manuscript to avoid overinterpretation. Statements implying definitive structural conservation or phylogenetic inference have been revised to more cautious descriptions such as “broadly conserved syntenic patterns” and “patterns generally consistent with established taxonomic relationships.” We now also explicitly note that these observations should be interpreted within the context of reference-guided scaffolding.

 Nevertheless, because the assemblies themselves were generated *de novo* and the comparative analyses were based on orthologous gene annotations rather than direct reference-guided genome alignment, we believe the analyses still provide informative comparative insights into genome organization among the examined *Betta* species.

 6. Given the authors are using gene models for their comparative analysis (not sequences directly verified with molecular methods), the authors should report support for identified protein insertions with primary data (e.g. insertion spanning long reads, depth of coverage at the insertion site, etc.). **Response:**

 Thank you for this important suggestion. To validate the inferred insertion identified from the gene model, we mapped Oxford Nanopore long reads back to the assembled genome. Coverage analysis revealed consistent read depth across the insertion locus (~70–88×), with no evidence of local coverage collapse or assembly breakpoints, thereby supporting the authenticity of the insertion. In addition, long-read alignments spanning the insertion region were visually inspected using Integrative Genomics Viewer and have been included in the Supplementary Materials deposited in figshare (DOI: https://doi.org/10.6084/m9.figshare.31189546.v2 ). The following supplementary materials have been added:

 Supplementary Figure S3. Validation of the CSF1R1 insertion using Oxford Nanopore long-read alignments. Coverage depth across the insertion locus remained consistent (~70–88×), and multiple long reads continuously spanned the insertion region, supporting the authenticity of the predicted insertion and excluding local assembly artifacts.

 Supplementary File S4. Coverage depth of the CSF1R1_1 region based on Oxford Nanopore long-read alignments.

 7. In the discussion, the authors make claims about purifying selection, rate of gene evolution, lineage specific selection, etc.. These are inferences that can only be drawn with population genetic data. These claims are improper for the data presented (e.g. assemblies of single individuals) and should be removed or qualified as such.

**Response:**

 Thank you for this valuable comment. We agree that strong evolutionary inferences such as purifying selection, lineage-specific selection, or adaptive evolution cannot be conclusively established from genome assemblies derived from single individuals without population-level genomic data. To avoid overinterpretation, we have revised the Discussion section to use more cautious and descriptive language that better reflects the scope of the current dataset.

 The following changes were made in the revised manuscript:

- “...reflecting strong purifying selection on this essential signaling pathway”
  was revised to
  “...reflecting high sequence conservation and potential evolutionary constraints on this essential signaling pathway.”
- “Similarly, the non-concordant evolution of MC1R, despite its variable role in teleost pigmentation, suggests relaxed constraint or functional innovation within the *Betta* lineage.”
  was revised to
  “Similarly, the non-concordant evolution of MC1R, despite its variable role in teleost pigmentation, is consistent with high sequence divergence that may warrant future investigation into potential regulatory or functional divergence within the *Betta* lineage.”
- “...may hint at ongoing lineage-specific regulatory or structural adaptations...”
  was revised to
  “...may hint at lineage-specific regulatory or structural variations.”
- “...potentially linked to ecological adaptations or evolutionary divergence...”
  was revised to
  “...potentially linked to evolutionary divergence among these lineages. However, population-level genomic data will be required to definitively connect these structural variations to specific ecological adaptations.”

These revisions were made throughout the Discussion section to ensure that the interpretations remain appropriately conservative and fully supported by the available data.

**Additional comments**

 1. The manuscript would be strengthened by reporting metrics from initial k-mer profiling with GenomeScope2. These could be reported in the form of the annotated k-mer distribution plot generated by GenomeScope2.

**Response:**

 Thank you for the suggestion. We have added the 21-mer profiling analysis to Figure 3. Accordingly, we have revised the figure caption as follows:

 “Figure 3. 21-mer–based genome size estimation (upper panels) and summary of genome assembly characteristics (lower panels) for *Betta burdigala*, *Betta chloropharynx*, and *Betta schalleri*.”

 2. The authors do not describe any methods for decontamination (either technical or biological). If these were not performed, this should be explicitly noted. If they were, what methods were employed and if they were pre-assembly or post assembly should be specified.

**Response:**

 Thank you for noticing. We apologize for not mentioning the contamination screening method used for our assembly. Prior to assembly, we conducted adapter contamination screening using Porechop_ABI, and post-assembly, we screened for foreign contamination using FCS-GX. We have now included the contamination screening details in the methods section under the “Genome Assembly and Annotation” subsection.

*“* *Adapter contamination screening was performed using Porechop_ABI prior to assembly”*

 And *“* *Foreign contamination was screened and removed using NCBI FCS-GX prior to scaffolding”*

 3. The authors do make publicly available the raw data, genome assemblies, and annotations. I would also recommend additionally submitting the repeats to public databases such as Dfam or FishTEDB.

**Response:**

 Many thanks for the suggestion. We plan to submit it to the DFAM database for the near future.

# Reviewer report: Reviewer response for version 1

**DOI:** 10.5256/f1000research.192703.r461103

**Published:** 2026-02-26

**Authors:** Bingpeng Xing

**Affiliations:** 1 Third Institute of Oceanography Ministry of Natural Resources, Xiamen, China

This manuscript focuses on three endemic Betta fishes from Bangka Island— *Betta burdigala, B. chloropharynx, and B. schalleri—*conducting an integrated study of meristic analysis and whole-genome assembly, and it makes the related sequencing data and assembly results publicly available. For endemic fishes of Bangka Island peatland wetlands, this study provides the first genomic resources and has potential value for conservation genomics and species evolution research.

 Overall, the data release is relatively complete, and the assembly contiguity and BUSCO completeness are high, meeting the basic requirements of a resource-type genome paper. However, the manuscript still needs strengthening in the following aspects:

 1.    Reference genome–guided scaffolding may affect structural and collinearity conclusions. The authors used RagTag together with the Betta splendens reference genome for scaffold construction. This approach is feasible for generating chromosome-scale assemblies, but it may also introduce structural bias to some extent, making the assembly closer to the reference genome structure. The conclusions in the current manuscript such as “high collinearity,” “closer phylogenetic relationships,” and “highly conserved structure” are all based on this scaffolding result; therefore: it is recommended to clearly state the potential limitations of reference-guided scaffolding in the Methods and Discussion; and clarify whether the collinearity analysis was based on the original contigs or the reference-guided scaffolds.

 2.    The sample size is limited, and over-inference at the species level should be avoided. The morphological analysis includes only three individuals per species, and genome sequencing includes only one individual per species. This is acceptable for a resource-type paper, but inferences in the following aspects should be more cautious: patterns of interspecific differences; gene duplication dynamics; specific gene insertion events; and inference of evolutionary divergence time.

 3.    The two “large insertions” in CSF1RA require stronger supporting evidence. The manuscript reports that CSF1RA in B. burdigala contains two large amino-acid insertions and speculates that they may affect pigment regulation. This result has potential novelty but currently lacks the following validation: long-read coverage; read-level support at the insertion boundaries; whether there are low-complexity or repetitive-sequence artifacts; and whether assembly or annotation errors have been excluded.

 4.    Evidence from a single-gene phylogenetic analysis is limited. The authors constructed a phylogenetic tree using the single gene CSF1RA and compared it with the NCBI taxonomy tree. A single-gene tree can only serve as a consistency check and cannot provide strong support for phylogenetic relationships.

 5.    Genome quality assessment metrics can still be strengthened. The manuscript reports N50, scaffold number, BUSCO, GC content, and gap percentage; these metrics are relatively basic. It is recommended to add sequencing depth (estimated coverage); read length N50 or mean read length; k-mer completeness analysis; assembly quality value (QV); and contamination detection methods (if any). Adding 2–3 of the above metrics can significantly improve confidence in the assembly.

 6.    The background statements in the Introduction needs revision. There are unclear and illogical sentences in the Introduction, such as the description of “16 years” and “2 species in Thailand and 9 species in Indonesia,” which is confusing. Rewrite the relevant paragraphs to make the logic clearer; describe more accurately recent progress in taxonomy and diversity research of the genus Betta; and standardize IUCN status as “Critically Endangered (CR)” or “Endangered (EN)” with clear citations.

 7.    The Latin name format is not standardized. The manuscript should be unified: genus and species names in italics, such as Betta burdigala; abbreviated form B. burdigala; in “Betta spp.” only Betta in italics and “spp.” not in italics; and the family name Osphronemidae not in italics. There are many cases of missing italics or inconsistency, and a full-text unified check is recommended.

 8.    There are many spelling and language errors, including but not limited to: criticaly → critically, Genome assembljies → assemblies, inconsistent spelling such as chlorpharynx / chloropharynx, etc. A comprehensive language check is recommended.

Is the work clearly and accurately presented and does it cite the current literature?

Partly

If applicable, is the statistical analysis and its interpretation appropriate?

Partly

Are all the source data underlying the results available to ensure full reproducibility?

Yes

Is the study design appropriate and is the work technically sound?

Partly

Are the conclusions drawn adequately supported by the results?

Partly

Are sufficient details of methods and analysis provided to allow replication by others?

Partly

Reviewer Expertise:

marine biology，genome，fish，genetic，DNA，eDNA

I confirm that I have read this submission and believe that I have an appropriate level of expertise to confirm that it is of an acceptable scientific standard, however I have significant reservations, as outlined above.

# Response

**Published:** 2026-05-18

**Authors:** Luthfi Nurhidayat

**Affiliations:** Faculty of Biology Universitas Gadjah Mada, Indonesia

1.    Reference genome–guided scaffolding may affect structural and collinearity conclusions. The authors used RagTag together with the Betta splendens reference genome for scaffold construction. This approach is feasible for generating chromosome-scale assemblies, but it may also introduce structural bias to some extent, making the assembly closer to the reference genome structure. The conclusions in the current manuscript such as “high collinearity,” “closer phylogenetic relationships,” and “highly conserved structure” are all based on this scaffolding result; therefore: it is recommended to clearly state the potential limitations of reference-guided scaffolding in the Methods and Discussion; and clarify whether the collinearity analysis was based on the original contigs or the reference-guided scaffolds.

**Response:**

 Thank you for this thoughtful comment. We agree that reference-guided scaffolding may introduce structural bias toward the reference genome and that this limitation should be clearly acknowledged when interpreting large-scale synteny and collinearity patterns.

 In our study, the primary genome assemblies were first generated *de novo* from Oxford Nanopore long reads. The resulting contigs were subsequently scaffolded using RagTag with the *Betta splendens* genome as a guide to improve chromosome-scale continuity. We acknowledge that this scaffolding strategy may partially influence large-scale structural organization and therefore could increase apparent similarity to the reference genome.

 To clarify this point, we have revised the Result and Discussion sections to explicitly state the potential limitations associated with reference-guided scaffolding. We also clarified that the collinearity analyses were conducted using annotated protein-coding genes and orthologous gene order on the scaffolded assemblies, rather than direct whole-genome nucleotide alignments. Therefore, the inferred syntenic relationships primarily reflect conservation of gene content and relative gene order rather than exact chromosome-scale structural identity.

 In addition, we have softened the interpretation throughout the Discussion section to avoid overstatement. For example, statements such as “significant genome conservation,” “highly conserved structure,” and “closer evolutionary relationship” were revised to more cautious wording including “broadly conserved syntenic patterns,” “comparatively higher levels of shared gene order,” and “patterns generally consistent with existing taxonomic relationships.” We also now emphasize that the observed collinearity patterns should be interpreted as comparative genomic signals within the context of reference-guided scaffolding, rather than definitive evidence of fully conserved chromosomal architecture.

 Nevertheless, because the assemblies were initially generated *de novo* and the synteny analysis was based on annotated orthologous genes rather than direct reference-guided genome alignment, we believe the analyses still provide informative comparative insights into genome organization among the examined *Betta* species.

 2.    The sample size is limited, and over-inference at the species level should be avoided. The morphological analysis includes only three individuals per species, and genome sequencing includes only one individual per species. This is acceptable for a resource-type paper, but inferences in the following aspects should be more cautious: patterns of interspecific differences; gene duplication dynamics; specific gene insertion events; and inference of evolutionary divergence time.

**Response:**

 Thank you for this important comment. We agree that the limited sample size, particularly the use of a single sequenced individual per species, requires cautious interpretation of species-level inferences. Our primary objective in this study was to generate foundational genomic resources for these threatened *Betta* species rather than to make definitive population-level evolutionary conclusions.

 In response, we revised the manuscript to avoid overinterpretation by moderating statements related to interspecific differences, gene duplication patterns, insertion events, and evolutionary divergence. We replaced overly definitive wording with more cautious expressions such as “is consistent with,” “may represent,” or “suggests potential variation,” where appropriate.

 We also added statements in the last paragraph of Discussion acknowledging that the assemblies represent single individuals and therefore may not capture the full extent of intraspecific genomic variation. Similarly, we noted that the limited number of specimens used for morphological analyses constrains broader species-level interpretations. However, we note that the examined *Betta* species are highly endemic and threatened taxa, making specimen collection ethically and logistically challenging. Within this context, the use of three specimens per species for morphological analyses and one individual per species for reference genome generation is consistent with many genomic resource studies involving rare or endangered organisms.

 We believe these revisions provide a more balanced interpretation while preserving the value of the genomic resources presented in this study.

 3.    The two “large insertions” in CSF1RA require stronger supporting evidence. The manuscript reports that CSF1RA in B. burdigala contains two large amino-acid insertions and speculates that they may affect pigment regulation. This result has potential novelty but currently lacks the following validation: long-read coverage; read-level support at the insertion boundaries; whether there are low-complexity or repetitive-sequence artifacts; and whether assembly or annotation errors have been excluded.

**Response:**

 Thank you for this important suggestion. To validate the inferred insertion identified from the gene model, we mapped Oxford Nanopore long reads back to the assembled genome. Coverage analysis revealed consistent read depth across the insertion locus (~70–88×), with no evidence of local coverage collapse or assembly breakpoints, thereby supporting the authenticity of the insertion. In addition, long-read alignments spanning the insertion region were visually inspected using Integrative Genomics Viewer and have been included in the Supplementary Materials deposited in figshare (DOI: https://doi.org/10.6084/m9.figshare.31189546.v3 ). The following supplementary materials have been added:

 Supplementary Figure S3. Validation of the CSF1R1 insertion using Oxford Nanopore long-read alignments. Coverage depth across the insertion locus remained consistent (~70–88×), and multiple long reads continuously spanned the insertion region, supporting the authenticity of the predicted insertion and excluding local assembly artifacts.

 Supplementary File S4. Coverage depth of the CSF1R1_1 region based on Oxford Nanopore long-read alignments.

 4.    Evidence from a single-gene phylogenetic analysis is limited. The authors constructed a phylogenetic tree using the single gene CSF1RA and compared it with the NCBI taxonomy tree. A single-gene tree can only serve as a consistency check and cannot provide strong support for phylogenetic relationships.

**Response:**

 Thank you for this valuable comment. We agree that phylogenetic inference based on a single gene has limited power for resolving species-level evolutionary relationships and should not be interpreted as definitive phylogenetic evidence. In our study, the CSF1RA phylogeny was intended primarily as a gene-level comparative analysis and a consistency check against the established taxonomy, rather than as a comprehensive reconstruction of species relationships. To avoid overinterpretation, we have revised the relevant sections of the manuscript to clarify the purpose and limitations of the single-gene analysis. We now explicitly state that the CSF1RA tree is presented to illustrate evolutionary conservation and relative clustering patterns among related taxa, whereas accepted species relationships remain based on established taxonomy and previous phylogenomic studies.

 In the Results section, we revised the sentence:

 “We found that the CSF1RA phylogenetic tree closely resembles the phylogenetic tree of the nine fish species compared based on NCBI taxonomic data”

 to:

 “Gene-based phylogenetic analyses showed that the CSF1RA tree exhibited clustering patterns broadly consistent with the established taxonomic relationships among the nine fish species based on NCBI taxonomy.”

 We also added the following statement to the Results section:

 “We note that these analyses are based on single genes and therefore are intended primarily for comparative assessment of gene-level evolutionary patterns rather than for robust species-level phylogenetic inference.”

 In addition, we revised the Discussion section to reduce overinterpretation of the single-gene phylogenetic analyses and to present the evolutionary implications more cautiously.

 5.    Genome quality assessment metrics can still be strengthened. The manuscript reports N50, scaffold number, BUSCO, GC content, and gap percentage; these metrics are relatively basic. It is recommended to add sequencing depth (estimated coverage); read length N50 or mean read length; k-mer completeness analysis; assembly quality value (QV); and contamination detection methods (if any). Adding 2–3 of the above metrics can significantly improve confidence in the assembly.

**Response:**

 Thank you for this valuable suggestion. We agree that including additional assembly quality metrics would strengthen confidence in the genome assemblies. In response, we have revised the manuscript to incorporate additional sequencing and quality-control information.

 First, we updated the *Genomic DNA extraction and sequencing* subsection in the Methods section. The original text:

 “The genome sequence data for *Betta burdigala* includes 16.38 million reads and 37.39 gigabases. For *Betta chloropharynx*, the data comprises 14.97 million reads and 57.97 gigabases. Meanwhile, *Betta schalleri* has 11.83 million reads and 23.75 gigabases.”

 has been revised to:

 “The genome sequence data for *Betta burdigala* comprised 16.38 million reads totaling 37.39 Gb, with a read N50 of 5.23 kb, corresponding to an estimated genome coverage of approximately 74.8–83.1× assuming a genome size of 450–500 Mb. For *Betta chloropharynx*, sequencing generated 14.97 million reads totaling 57.97 Gb, with a read N50 of 7.75 kb, representing an estimated coverage of approximately 115.9–128.8×. Meanwhile, *Betta schalleri* produced 11.83 million reads totaling 23.75 Gb, with a read N50 of 5.5 kb, corresponding to an estimated genome coverage of approximately 47.5–52.8×.”

 These additions provide both sequencing depth estimates and read-length statistics requested by the reviewer.

 Second, we updated the *Genome assembly and annotation* subsection to include contamination screening and preprocessing procedures that were inadvertently omitted from the original manuscript. Specifically, we added the following statements:

 “Adapter contamination screening was performed using Porechop_ABI prior to assembly.”

 “Foreign contamination was screened and removed using NCBI FCS-GX prior to scaffolding.”

 Finally, we also incorporated the 21-mer–based genome size estimation analysis into the revised Figure 3, which provides an additional k-mer–based assessment supporting assembly completeness and consistency with the final assembly sizes.

 6.    The background statements in the Introduction needs revision. There are unclear and illogical sentences in the Introduction, such as the description of “16 years” and “2 species in Thailand and 9 species in Indonesia,” which is confusing. Rewrite the relevant paragraphs to make the logic clearer; describe more accurately recent progress in taxonomy and diversity research of the genus Betta; and standardize IUCN status as “Critically Endangered (CR)” or “Endangered (EN)” with clear citations.

**Response:**

 Thank you for the suggestion. We have revised the statement:

 “The number of endemic wild Betta distribution and diversity of Southeast Asia has been identified for 16 years in Thailand (2 species) and Indonesia (9 species) ^1-5^”

 to

 “Indonesia harbors nine wild Betta species classified as critically endangered and seven species categorized as endangered ^1-3^. In comparison, Thailand contains two critically endangered wild Betta species and one endangered species ^4,5^.”

 7.    The Latin name format is not standardized. The manuscript should be unified: genus and species names in italics, such as Betta burdigala; abbreviated form B. burdigala; in “Betta spp.” only Betta in italics and “spp.” not in italics; and the family name Osphronemidae not in italics. There are many cases of missing italics or inconsistency, and a full-text unified check is recommended.

**Response:**

 Thank you for this valuable comment, and we apologize for the inconsistencies in the formatting of scientific names throughout the original manuscript. We agree that standardized formatting of Latin names is important for taxonomic clarity and overall manuscript readability.

 In response, we conducted a full-text review of the manuscript to standardize the formatting of scientific names throughout the text, figures, tables, and supplementary materials. Genus and species names are now consistently italicized (e.g., *Betta burdigala*), abbreviated species names are formatted correctly (e.g., *B.* burdigala), and in expressions such as *Betta* spp., only the genus name is italicized while “spp.” remains in standard font. In addition, family names such as Osphronemidae are now consistently presented in non-italicized format.

 We also corrected multiple instances of missing italics and formatting inconsistencies throughout the manuscript to ensure conformity with standard zoological nomenclature conventions.

 8.    There are many spelling and language errors, including but not limited to: criticaly → critically, Genome assembljies → assemblies, inconsistent spelling such as chlorpharynx / chloropharynx, etc. A comprehensive language check is recommended.

**Response:**

 Thank you for pointing this out. We appreciate the reviewer’s careful reading of the manuscript. In response, we conducted a comprehensive language and spelling revision throughout the manuscript to correct typographical errors, improve grammar, and ensure consistency in terminology and species names.

 Specifically, spelling errors such as “criticaly” to “critically,” “Genome assembljies” to “genome assemblies,” and inconsistent species naming (e.g., “chlorpharynx” to “chloropharynx”) were corrected throughout the text. We also carefully reviewed formatting consistency, including the standardized use of italicization for scientific names.
